# Cystic echinococcosis in cattle and sheep caused by *Echinococcus granulosus *sensu stricto genotypes G1 and G3 in the USA

**DOI:** 10.1186/s13071-024-06192-x

**Published:** 2024-03-14

**Authors:** Jeba R. J. Jesudoss Chelladurai, Theresa A. Quintana, William L. Johnson, Carrie Schmidt, Daniel Righter, Erin Howey

**Affiliations:** 1 Department of Diagnostic Medicine/Pathobiology, College of Veterinary Medicine, Kansas State University, Manhattan, KS USA; 2 Pathology Branch, Eastern Laboratory, Office of Public Health Science, Food Safety and Inspection Service, United State Department of Agriculture, Athens, GA USA

**Keywords:** Echinococcus granulosus, Ruminants, USA, Haplotype, Genotype, Hydatid cyst

## Abstract

**Background:**

Endemic domestic dog-ruminant cycles and human cystic echinococcosis caused by *Echinococcus granulosus* have been sporadically reported in the United States. However, there is a paucity of molecular data describing the genotypes and haplotypes of this important cestode in domestic ruminant hosts.

**Methods:**

Ninety-four cysts from the lungs and/or livers of slaughtered beef cattle (76 samples), dairy cows (five samples) and sheep (13 samples) were collected from abattoirs in four states of the USA. Samples were genotyped at two mitochondrial loci, *cox1* and *nad5.* Sequences were used to determine species, genotypes and haplotypes using median joining networks and Bayesian phylogenetic analyses. Cyst fertility was assessed in hematoxylin and eosin-stained sections. Additionally, previously reported autochthonous *E. granulosus* infections in the USA in various hosts were mapped.

**Results:**

Based on *cox1* sequences obtained from 94 cysts, 89 (94.7%) were identified as *E. granulosus* G1/G3, while five (5.3%) were *Taenia hydatigena. Taenia hydatigena* were only isolated from sheep. Based on *nad5* sequences obtained from 89 hydatid cysts, 96.6% and 3.4% belonged to *E. granulosus *sensu stricto genotypes G1 and G3 respectively. Two haplotypes were found among *E. granulosus cox1* sequences, neither of which was geographically unique. Six haplotypes were found among *nad5* sequences in genotype G1, of which five were novel, while one haplotype was found in genotype G3. In the concatenated *cox1-nad5* dataset, seven haplotypes were identified, of which six were geographically unique. All cysts from cattle were non-fertile. Four cysts from sheep were fertile.

**Conclusions:**

All genotyped samples belonged to *E. granulosus *s.s. This is the first study to our knowledge to confirm the presence of genotypes G1 and G3 in domestic cattle and sheep intermediate hosts in the USA and provide data for future diagnostic and epidemiological studies. Sequences have been deposited in GenBank (*cox1* sequences: OR398494-OR398496, *nad5* sequences: OR400695-OR400702).

**Graphical Abstract:**

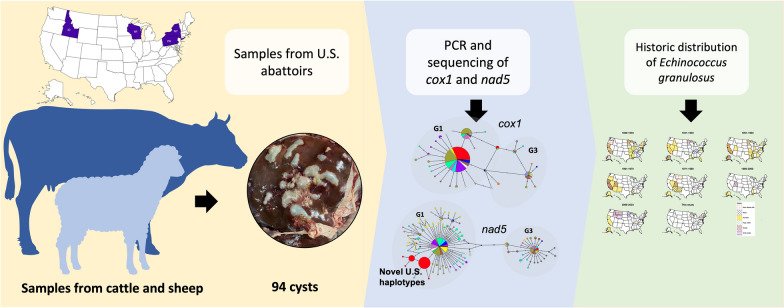

**Supplementary Information:**

The online version contains supplementary material available at 10.1186/s13071-024-06192-x.

## Background

Cystic echinococcosis (CE), also known as hydatid disease or hydatidosis, is caused by zoonotic cestodes in the species complex *Echinococcus granulosus *sensu lato (Taeniidae: Cestoda). The life cycle of *E. granulosus* can be domestic or sylvatic and typically involves a carnivore and herbivore. Humans may become accidental dead-end hosts and suffer from CE [1]. In humans, the World Health Organization (WHO) Foodborne Disease Burden Epidemiology Reference Group estimates that CE costs 184,000 disability-adjusted life years globally each year [2]. In production animals, annual losses are estimated at $2 billion globally because of losses in carcass weight, milk production, fecundity and wool/hide production [3]. Livestock production losses within the USA are not well documented. However, they are likely negligible compared to global losses based on the low number of samples collected during routine slaughter inspection by USDA Food Safety and Inspection Service (FSIS) Public Health Veterinarians. Suspected CE cases are collected by FSIS veterinarians for histological examination and confirmation of etiology. Despite the large number of studies performed globally to understand the prevalence and distribution of CE [4], the epidemiology of CE in animals and humans in North America is understudied [3, 5]. Specifically in the US, autochthonous human CE was reported in 38 cases between 1856 and 1956 [6] and 123 cases between 1900 and 1974 [7] and identified as the cause of death in 41 humans between 1990 and 2007 [8]. Similar to production losses, the epidemiology in domestic animals has not been well characterized in the USA beyond sporadic reports presumably because of the negligible socioeconomic burden.

*Echinococcus granulosus* obligately requires two hosts—a canid definitive host and an intermediate host—to complete its life cycle. Humans and other intermediate hosts acquire the infection through the ingestion of *E. granulosus* eggs shed in the feces of infected canine hosts. In intermediate hosts, the metacestode form of the parasite, called a hydatid cyst, occurs. The cysts are only infectious to the canid definitive hosts and not to humans. In the USA, dogs [9–12], gray wolves (*Canis lupus*) [13, 14] and coyotes (*Canis latrans*) [14–16] can serve as definitive hosts and sources of infection. Domestic intermediate hosts that support the life cycle in the USA include sheep [9], pigs [17, 18] and cattle [19, 20]. Wild ungulates such as elk (*Cervus elaphus*), mule deer (*Odocoileus hemionus*) and mountain goats (*Oreamnos americanus*) [13] play a role in maintaining a sylvatic life cycle. Analyzing hydatid cysts in intermediate hosts can be a useful surrogate to understand the molecular epidemiology of the cestode in both the domestic and sylvatic cycle.

The taxonomy of *E. granulosus* has been revised based on analyses of mitochondrial gene sequences. Eight genotypes are now recognized [21]. These include *Echinococcus granulosus * s.s. (G1-3, with G2 recognized as a microvariant of G3; G Omo), *E. equinus* (previously G4), *E. ortleppi* (previously G5) and *E. canadensis* (G6/G7, G8, G10) [21, 22]. Of these, *E. granulosus* s.s. is frequently identified in molecularly confirmed human CE cases in endemic parts of the world [23]. It is important to know the prevalent genotypes and molecular epidemiology of *Echinococcus* infections in different geographical areas to characterize the reservoir(s) for this parasite and to gauge the risk of transmission within different regions.

There is a marked lack of publicly available molecular data from *E. granulosus* isolates from the USA, while isolates of the related species *Echinococcus multilocularis* has been characterized from a few locations in the country [24]. To understand the molecular epidemiology of *E. granulosus* in the USA, the aim of this study was to identify and genotype cysts from ruminants obtained from abattoirs in the USA.

## Methods

### Parasites and study area

As part of their routine inspection procedures, a total of 94 cysts were collected from the liver and lungs of domestic sheep and cattle by USDA FSIS Public Health Veterinarians for confirmation of suspected CE from abattoirs in four states (Idaho (81 cysts), New York (7 cysts), Pennsylvania ﻿(3 cysts) and Wisconsin (3 cysts)) in the USA between October 2021 and October 2022. Portions of suspected hydatid cysts were collected from 76 beef cattle, five dairy cows, four lambs and nine mature sheep by federal meat inspection personnel and were submerged in 10% neutral-buffered formalin for histologic evaluation and in 70% ethanol for molecular analyses. Samples were submitted to the Pathology Branch of USDA–FSIS. Upon arrival at the Eastern Laboratory (Athens, GA), the samples were routinely processed for histopathology, and all slides were stained with hematoxylin & eosin. Several slides were also routinely stained with periodic acid-Schiff (PAS). Morphological diagnosis and fertility were assessed by veterinary pathologists. Fertility terminology following the definitions outlined in [21] was used. Samples for molecular analyses were stored in 70% ethanol until shipment to the laboratory at Kansas State University College of Veterinary Medicine. All samples were anonymized for molecular analyses. All protocols were approved by the Institutional Biosafety Committee at Kansas State University (IBC-1638-VCS).

### DNA extraction, PCR and sequencing of *cox1* and *nad5*

Genomic DNA was extracted from cyst wall and cyst membranes of the 94 cysts and inflammatory masses using the Qiagen DNeasy tissue and blood kit (Valencia, CA) following the manufacturer’s protocol. DNA was eluted in nuclease-free water and stored at – 20 ℃.

A ~ 380-bp fragment of the mitochondrial cytochrome C oxidase subunit 1 (*cox1*) gene was amplified as previously described [25]. Since *cox1* is not deemed sufficiently consistent to differentiate between the G1 and G3 genotypes of *E. granulosus* s.s., a ~ 690-bp fragment of the mitochondrial NADH dehydrogenase subunit 5 (*nad5*) gene was amplified as previously described [26]. All amplifications were confirmed using agarose gel electrophoresis, and products were purified. Sanger sequencing of PCR amplicons was performed at Euro Fins Labs (Louisville, KY).

### Sequence analysis

Representative sequences from GenBank were obtained to compare data from this study to sequences worldwide. For *cox1* global analyses, 303 representative validated sequences of *E. granulosus* G1 and G3 genotypes from [26, 27] were used in addition to the sequences from this study. For *nad5* global analyses, 304 sequences from GenBank were obtained in addition to the sequences from this study. Multiple sequence alignment was performed with Multiple Alignment using Fast Fourier Transform (MAFFT) program [28]. Alignments were trimmed with the R package *microseq* version 2.1.5 [29] and with Block Mapping and Gathering with Entropy (BMGE) [30]. For *cox1* analyses, all sequences were trimmed to 323 bp. For *nad5* analyses, all sequences were trimmed to 670 bp. Haplotype analysis was performed with DnaSP version 6 [31], and median joining networks were created using Population Analysis with Reticulate Trees version 1.7 (PopART) [32]. Bayesian phylogenetic analysis of *nad5* sequences was conducted with MrBayes version 3.2.7 [33] and trees visualized with iTol version 5 [34]. Additionally, trimmed *cox1*-*nad5* sequences from this study and sequences from [27] were concatenated with the R package *phylotools* version 0.2.2 [35]. Median joining networks were created with PopART version 1.7.

To denote positions of nucleotide change in the multiple sequence alignment, USA haplotypes were compared to the *E. granulosus* mitochondrial genome GenBank accession no. AB786664 [36], which was used as the reference sequence in a previous study [26]. Visualization was performed with packages *ggmsa* version 1.4.0 [37] and *Biostrings* version 2.66.0 [38] in R version 4.2.2.

### Mapping autochthonous reports

Autochthonous cases and reports of *E. granulosus* s.l. in the USA in domestic and wildlife animal hosts and humans were obtained from published literature using keywords “Echinococcus” and “USA” in NCBI PubMed and Google Scholar databases. Reports were examined for unique geographical location (state of origin of case), host, time range (years) and the location of case acquisition (autochthonous or imported). Reports of *E. multilocularis* were excluded. Cases in humans diagnosed in the USA but acquired in another country during travel were also excluded. Unique references for each state and host are summarized in Additional file 1: Table S2. Primary references were often not available for older reports; therefore, secondary citations and reviews were used. Reports were collated in Microsoft Excel version 16.64 and mapped using packages *maps* version 3.4.1 [39], *ggplot2* version 3.4.4 [40]*, ggpubr* version 0.6.0 [41] and *fiftystater* version 1.0.1 [42] in R version 4.2.2. One autochthonous case of an infection in a horse [43] was not mapped.

## Results

### Sample collection and histopathological analysis

**Fig. 1:**
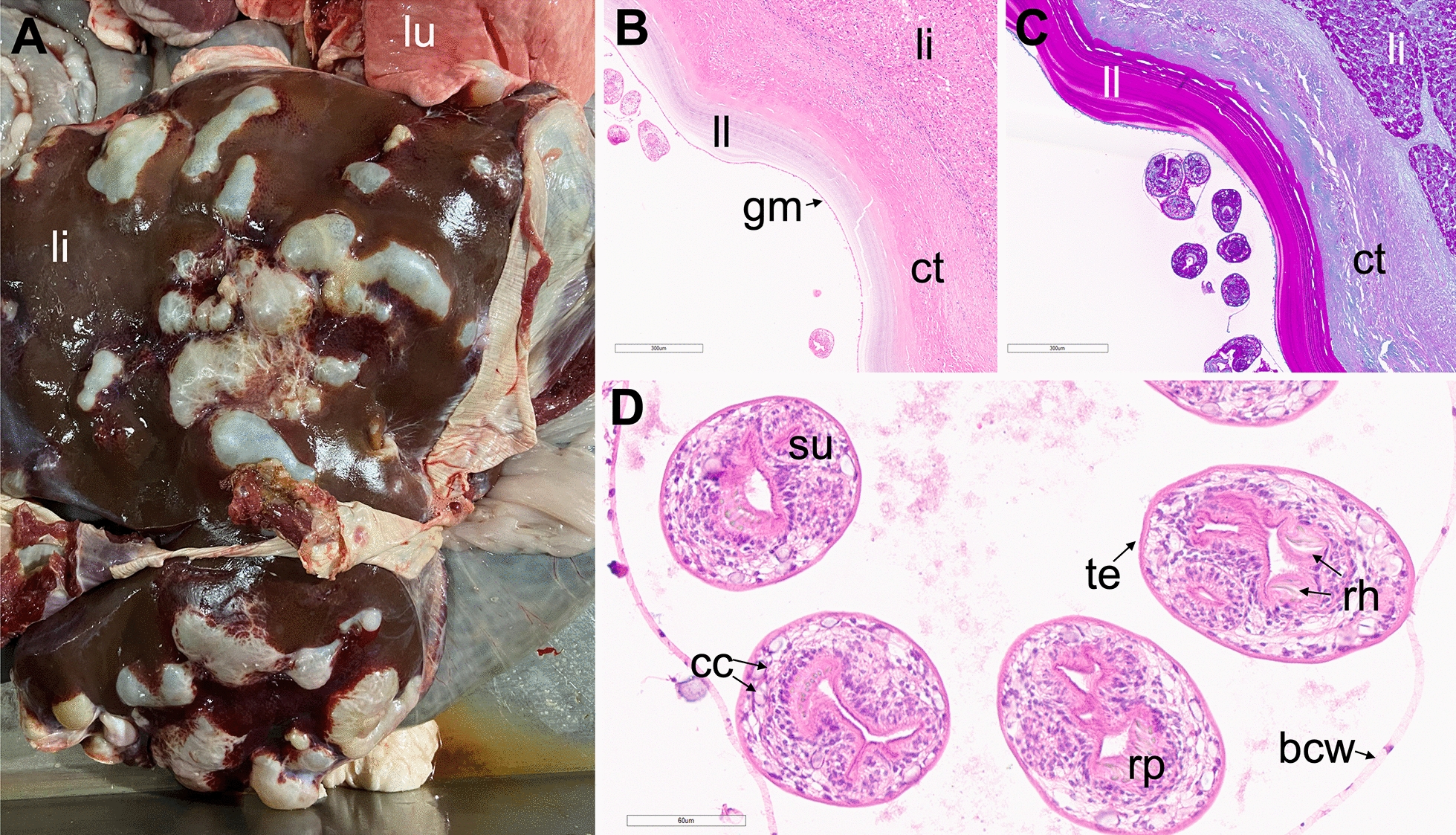
Liver and lung with hydatid cysts. **A** Liver with several hydatid cysts and lung with one hydatid cyst. **B** Hydatid cyst with protoscolices within the liver using hematoxylin-eosin staining. **C** Hydatid cyst with protoscolices within the liver using PAS technique. **D** High magnification (400×) of protoscolices surrounded by a brood capsule wall. *li* liver, *lu* lung, *ll* laminated layer, *ct* connective tissue, *gm* germinal membrane, *su* sucker, *cc* calcareous corpuscles, *te* tegument, *rh* rostellar hooks, *rp* rostellar pad, *bcw* brood capsule wall

From October 2021 to October 2022, we obtained 94 cysts and inflammatory masses from ruminant abattoirs in four states. Histopathology was performed on all fixed samples sent to the Eastern laboratory suspicious of hydatid cysts based on gross appearance (Fig. 1A). Of the 94 samples, 79 were confirmed as hydatid cysts histologically. Hydatid cyst confirmation by histology was based on the presence of a cyst with an eosinophilic, hyalinized, PAS-positive, laminated layer (Fig. 1B, C). In many cases, this laminated layer was lined internally by an intact to degenerate layer of epithelial cells (germinal membrane). All cases from cattle were non-fertile. In four cases isolated from sheep, protoscolices were found within the cysts (fertile). These protoscolices contained a thin, eosinophilic outer tegument, a loose parenchymous body admixed with scattered calcareous corpuscles and a branched digestive tract, with a rostellar pad and prominent rostellar hooks (Fig. 1D). In nearly all cases, cysts were surrounded by fibrosis and granulomatous inflammation, particularly in more chronic cases. Four cysts were severely degenerate and lacked a hyalinized membrane or other diagnostic features of hydatid cysts. These cysts were diagnosed as degenerate cysts (likely hydatidosis) since the etiology of the cyst could not be determined histologically. All cysts diagnosed histologically as hydatidosis or degenerate cysts (likely hydatidosis) were confirmed via PCR. Five cysts were diagnosed histologically as the metacestode form of *Taenia hydatigena*, namely Cysticercus tenuicollis. Diagnosis for all five cysts was confirmed by PCR. Six samples contained multiple concurrent lesions (e.g. bacterial pyogranuloma and hydatid cyst) where the hydatid cysts were not provided for histological evaluation.

### Genotypic and haplotypic analyses of *cox1* sequences

Samples for molecular analyses were submitted embedded in lung and liver parenchyma and ranged in size from 0.5 cm to 7 cm. Nucleotide sequences were obtained from 93 cysts (out of 94; 99%) for the *cox1* gene. *Cox1* could not be sequenced from one cyst. Based on *cox1* sequence analysis, 88 samples (out of 93; 94%) were identified as *E. granulosus* s.s. (G1/G3). Five cyst samples (out of 93; 5%) were identified as *T. hydatigena*. All identified *T. hydatigena* were isolated from mature sheep and lambs (Table 1). All three cyst samples tested from Wisconsin sheep were *T. hydatigena* based on *cox1* analysis.

**Table 1:** Results of molecular analysis of *cox1* and *nad5* sequences from 94 pulmonary and hepatic cysts isolated from ruminants in 4 USA states in this study

Host	Molecular ID			
	*Echinococcus granulosus* Genotype G1	*Echinococcus granulosus* Genotype G3	*Taenia hydatigena*	Total samples
Beef cow	73	3	–	76
Dairy cow	5	–	–	5
Lamb	1	–	3	4
Mature sheep	7	–	2	9

**Fig. 2:**
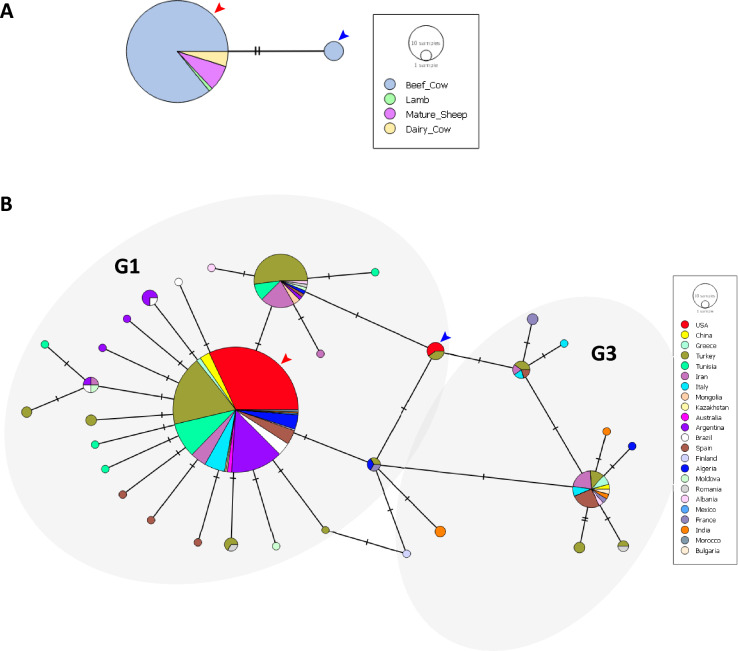
Networks of mitochondrial *cox1* gene. **A** Median joining haplotype network of *cox1* sequences from this study colored by host of origin. Hatch marks on lines connecting the nodes represent number of nucleotide changes. Arrowheads indicate the two haplotypes. **B** Median joining haplotype network of *cox1* sequences from this study and from GenBank colored by country of origin. Hatch marks on lines connecting the nodes represent number of nucleotide changes. Genotype clusters G1 and G3 are indicated. ﻿USA sequences are indicated by arrowheads

Haplotypic analysis of the *E. granulosus* s.s. *cox1* gene sequences from the USA samples in this study (88 sequences) revealed two haplotypes (Fig. 2A). Eighty-five samples from both cattle and sheep belonged to one haplotype (red arrowhead), while three samples from cattle belonged to the second haplotype (blue arrowhead). The haplotypes differed at two nucleotides, that is, positions 9853 and 9863 of the *cox1* gene, based on GenBank accession no. AB786664 [36]. Haplotypic diversity in the sample set was 0.067 (standard deviation 0.036) and nucleotide diversity was 0.00041 (standard deviation 0.00022).

Global analysis of partial *cox1* sequences with 391 sequences (including sequences from this study) from 23 countries revealed that the two haplotypes from this study were not unique (Fig. 2B). The most common haplotype (Fig. 2B; red arrowhead) from this study was found in the same cluster as others from 18 other countries, while the second haplotype (Fig. 2B; blue arrowhead) was in the same cluster as two sequences from Turkey [S20, haplotype TUR7; GenBank accession: MG672178.1 (G1) and S124, haplotype TUR26 GenBank accession: MG672195.1 (G1)] [26]. No unique *cox1* haplotypes were found in this study. There were 33 total haplotypes in the global dataset with a haplotypic diversity of 0.519 (standard deviation 0.029).

### Analysis of the mitochondrial *nad5* sequences

**Fig. 3:**
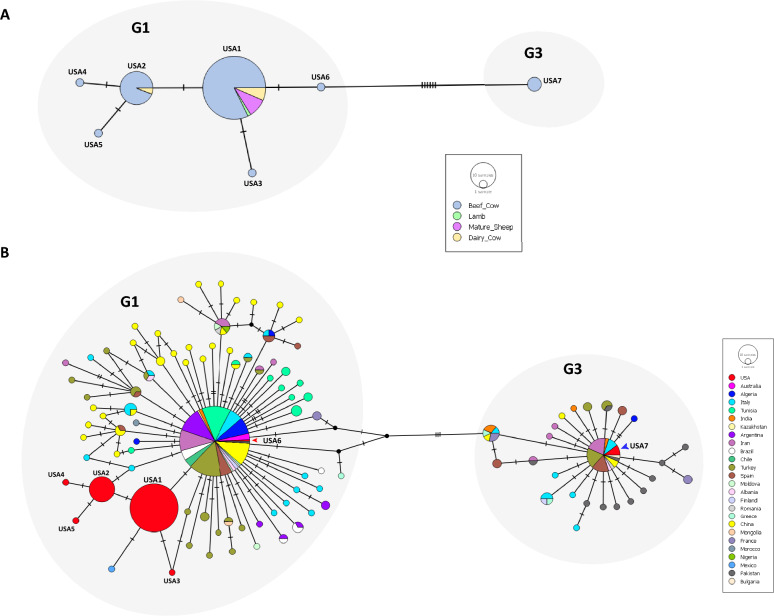
Networks of mitochondrial *nad5* gene. **A** Median joining haplotype network of *nad5* sequences from this study colored by host of origin. Hatch marks on lines connecting the nodes represent number of nucleotide changes. USA haplotypes are identified by numbered haplotype designations. **B** Median joining haplotype network of *nad5* sequences from this study and from GenBank colored by country of origin. Dark circles represent putative undetected haplotypes. Hatch marks on lines connecting the nodes represent number of nucleotide changes. Genotype clusters G1 and G3 are indicated. USA haplotypes are identified by numbered haplotype designations

Nucleotide sequences were obtained from 86 *E. granulosus* cysts (out of 89; 97%) for the *nad5* gene. *Nad5* could not be sequenced from three small cysts. The *E. granulosus* species-specific *nad5* primers [26] did not amplify *nad5* from the five samples identified as *T. hydatigena* using *cox1* sequences. Based on *nad5* sequence analysis, 83 cysts (of 86; 96%) were identified as *E. granulosus* G1 genotype, while three cysts were identified as *E. granulosus* G3 genotype (out of 86; 4%) (Table 1, Fig. 3). Results of histological and molecular identification are presented in Additional file 1: Table S1.

### Novel haplotypes identified with *nad5* sequences

Haplotypic analysis of the *nad5* gene sequences from this study revealed the presence of seven haplotypes, of which six belonged to genotype G1 and one to genotype G3 (Fig. 3A). These were designated “USA”1–7 based on frequency of occurrence. Eighty-three samples from both cattle and sheep were distributed among the six haplotypes within G1 (Table 2). Three samples from cattle were found in haplotype G3. Haplotype USA1 (G1) was the most common haplotype (62 cysts out of 86; 72%). Haplotype USA2 (G1) was the second most common (17 cysts; 20%), while one cyst (1%) each belonged to haplotypes USA 3, 4, 5 and 6 (G1). Three cysts (3%) belonged to haplotype USA7 (G3). Overall, haplotypes from this study differed at 12 nucleotides from the *nad5* gene in GenBank accession no. AB786664 [36], that is, at positions 736, 758, 781, 783, 972, 974, 984, 1035, 1074, 1123, 1371 and 1380 (Additional file 1: Fig. S1, Table 3). Haplotype USA7 (genotype G3) differed from USA6 (genotype G1) at six nucleotides. Haplotypic diversity in the sample set was 0.445 (standard deviation 0.057) and nucleotide diversity was 0.00137 (standard deviation 0.00039).

**Table 2:** Distribution of *nad5* haplotypes of *Echinococcus granulosus *sensu stricto in the samples analyzed in this study by host and state of origin

State and haplotype (Genotype) (GenBank accession)	Host			
	Beef cow	Dairy cow	Lamb	Mature sheep
Idaho				
Haplotype USA1 (G1) (OR400695)	52	4	–	–
Haplotype USA2 (G1) (OR400697)	16	1	–	–
Haplotype USA3 (G1) (OR400698)	1	–	–	–
Haplotype USA4 (G1) (OR400699)	1	–	–	–
Haplotype USA5 (G1) (OR400700)	1	–	–	–
Haplotype USA6 (G1) (OR400701)	1	–	–	–
Haplotype USA7 (G3) (OR400702)	3	–	–	–
New York				
Haplotype USA1 (G1) (OR400696)	–	–	1	4
Pennsylvania				
Haplotype USA1 (G1) (OR400696)	–	–	–	1

**Table 3:** Nucleotide positions in the *nad5* gene differentiating *Echinococcus granulosus* haplotypes, with positions numbered according to the GenBank reference AB786664 (nucleotides 727–1396)

Genotypes	G1	G1	G1	G1	G1	G1	G1	G3
Haplotypes	AB786664	Haplotype USA1	Haplotype USA2	Haplotype USA3	Haplotype USA4	Haplotype USA5	Haplotype USA6	Haplotype USA7
Position								
**736**	A	A	A	A	A	**T**	A	A
**758**	G	G	G	G	G	G	G	**C**
**781**	A	A	A	A	A	A	A	**G**
**783**	C	C	C	**T**	C	C	C	**C**
**972**	A	A	**G**	A	**G**	**G**	A	A
**974**	T	**C**	**C**	**C**	**C**	**C**	T	T
**984**	G	G	G	G	G	**C**	G	G
**1035**	C	C	C	C	C	C	C	**T**
**1074**	C	**T**	**T**	**T**	**T**	**T**	**T**	**T**
**1123**	G	G	G	G	G	G	G	**A**
**1371**	A	A	A	A	A	A	A	**G**
**1380**	G	G	G	G	G	G	G	**A**

Nucleotide changes from the reference are indicated in bolded text

Global analysis of *nad5* sequences was performed with 390 sequences from 26 countries (Fig. 3B). There were 104 total haplotypes in the dataset with a haplotypic diversity of 0.852 (standard deviation 0.015) and nucleotide diversity of 0.00459 (standard deviation 0.00024). Of the six G1 haplotypes from this study, five were unique on the global scale. One haplotype (haplotype USA6) was found to be identical to the predominant global haplotype in genotype G1, a haplotype reported from 20 countries (including the USA; this study). Haplotype USA1 differed at only one nucleotide from a haplotype from a pig in Mexico (accession no. MG672259) [44]. Similarly, haplotypes USA2 and USA3 differed at one nucleotide each from a haplotype from cattle in Italy (Sardinia) (accession no. MT99398) and a haplotype from cattle in Turkey (accession no. MG672186) respectively. Additionally, the only G3 haplotype (haplotype USA7) was identical to the predominant global haplotype in genotype G3, a haplotype reported from 11 countries (including the USA; this study). No haplotypes unique to the USA were found in genotype G3 in this study.

### Phylogenetic evidence for *nad5* genotypes

**Fig. 4:**
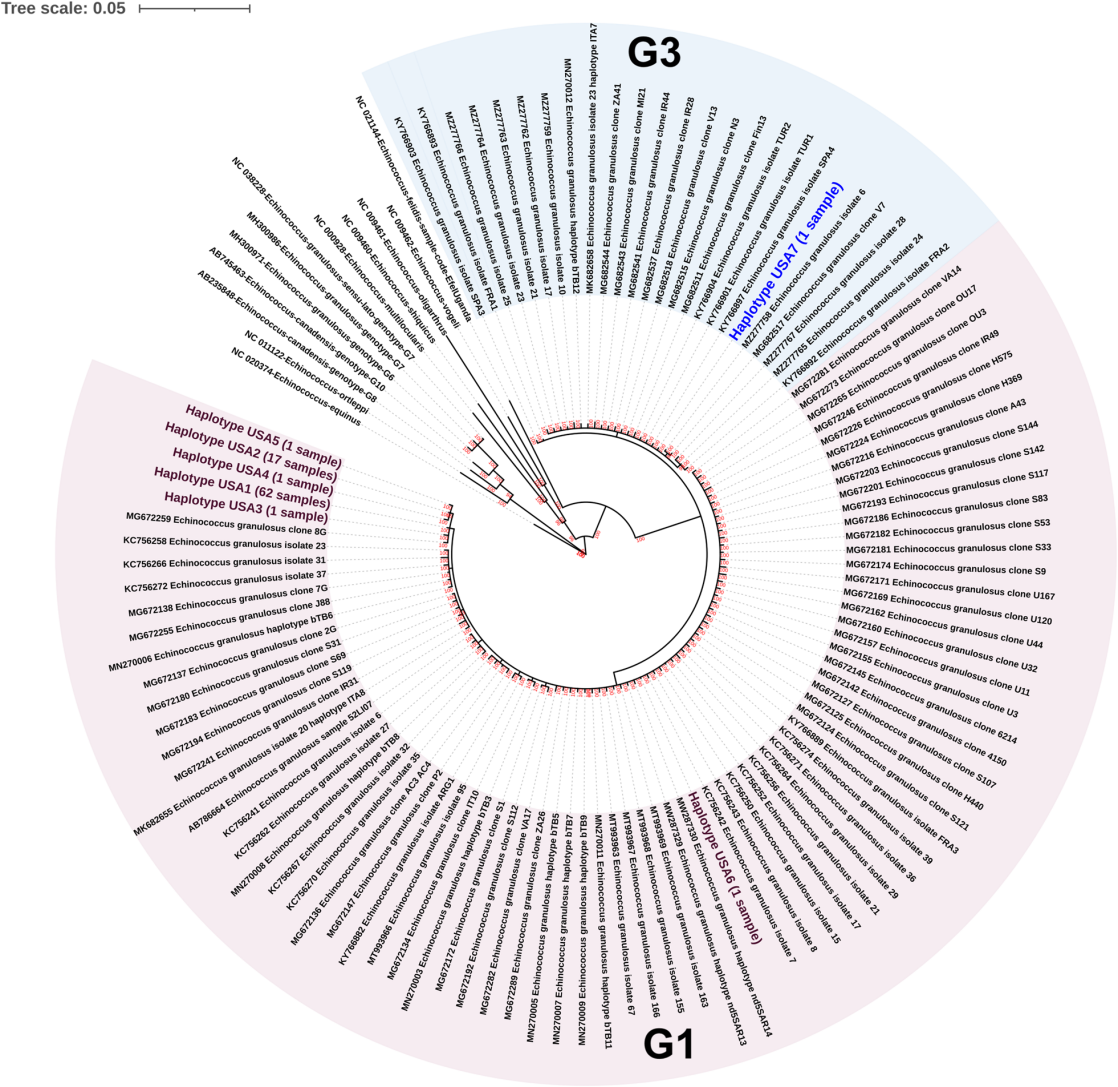
Bayesian phylogenetic analysis of *nad5* sequences representing unique haplotypes from this study and from GenBank. Clades of *Echinococcus granulosus* genotypes G1 and G3 are highlighted. Haplotypes identified in this study in genotypes G1 and G3 are colored. Bayesian posterior probabilities for branch support are displayed at each node

Bayesian phylogenetic analysis of unique *nad5* haplotypes from this study and GenBank sequences revealed that *E. granulosus* s.s. (G1/G3) formed a distinct clade (100% posterior probability) (Fig. 4). Within the G1/G3 clade, genotypes G1 and G3 were moderately supported with posterior probabilities of 60% and 77% respectively. Haplotype USA6 was found within the G1 clade with high statistical support (100% posterior probability), while unique haplotypes from this study (haplotypes USA1-5) were found in a moderately supported clade (posterior probabilities of 70%) along with the sequence from Mexico (accession no. MG672259). Haplotype USA7 was found within the G3 clade with high statistical support (100% posterior probability).

### Novel haplotypes identified with concatenated cox1-*nad5* sequences

**Fig. 5:**
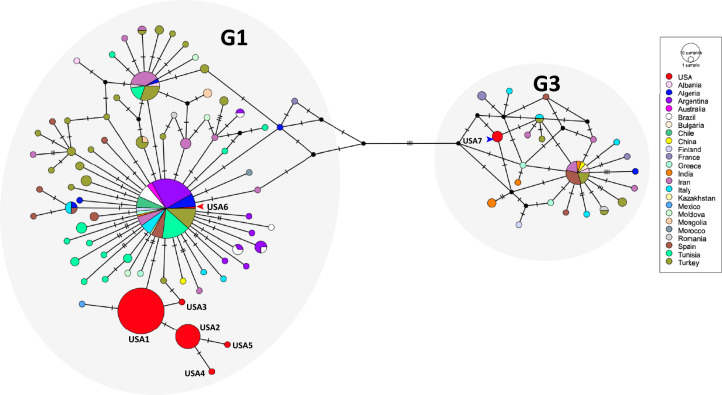
Networks of concatenated mitochondrial *cox1* and *nad5* genes. Median joining haplotype network of concatenated *cox1* and *nad5* sequences from this study and from Kinkar, [1, 26, 27, 44] colored by country of origin. Dark circles represent putative undetected haplotypes. Hatch marks on lines connecting the nodes represent number of nucleotide changes. Genotype clusters G1 and G3 are indicated. USA haplotypes matching *nad5* designations are identified

Global analysis of the concatenated dataset of *cox1-nad5* gene sequences contained 993 nucleotide sites with 335 sequences from 24 countries (Fig. 5). There were 94 total haplotypes in the dataset with a haplotypic diversity of 0.8765 (standard deviation 0.013). Of the six G1 haplotypes and single G3 haplotype from this study, five G1 and the single G3 haplotypes were unique to the USA. Within the G1 cluster, there was agreement with the *nad5* network within the available global data, with cox1/nad5 data for some countries/isolates being unavailable on GenBank. Additionally, the only G3 haplotype (haplotype USA7) was unique, differing at one nucleotide from a haplotype comprising of sheep isolates from Turkey (haplotype TUR42; accession MG682534) and Italy (haplotype ITA12; accession MG682521). Haplotype USA7 also differed at one nucleotide from two putative undetected haplotypes (dark circles in Fig. 5).

### Mapping reveals the temporal distribution of *E. granulosus*

**Fig. 6:**
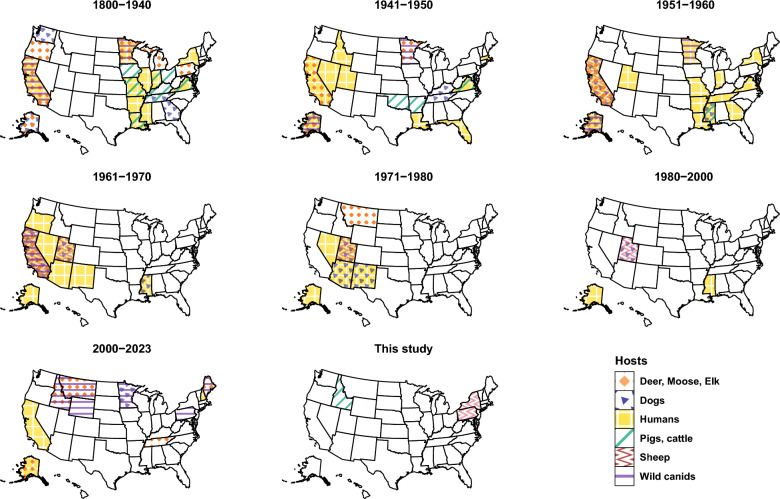
Map of the USA showing states with reports of autochthonous transmission of *Echinococcus granulosus*

Since the 1800s, autochthonous cases of *E. granulosus* s.l. have been reported from at least 32 states of the USA in all hosts (Fig. 6). Among definitive hosts, autochthonous cases in dogs have been reported from 11 states and cases in wild canids (coyotes and wolves) from eight states. Among domestic animal intermediate hosts, autochthonous cases in sheep have been reported from five states, and cases in pigs and cattle have been reported from 12 states. Among wild cervids, cases have been reported from 10 states. Humans may also serve as accidental intermediate hosts; autochthonous human cases have been reported from 22 states. All efforts were taken to report only autochthonous human cases, as reported in the original reports. However, it should be noted that sources and times of infection can be difficult to determine and can be protracted for up to 10–15 years before symptoms appear and diagnosis can be reached.

## Discussion

CE can result in devastating lesions and disease in humans and intermediate domestic animal hosts. *Echinococcus* spp. are understudied in the USA, and the epidemiology of CE in humans and animals in the country is not completely known [3]. Based on historical reports in the USA, it is evident that CE has a domestic life cycle involving domestic dogs and livestock, with humans serving as dead-end hosts. Thus, the life cycle of *E. granulosus* s.s. in the USA is expected to be animal-centric as in other parts of the world. In the present study, we demonstrate the occurrence of *E. granulosus* s.s. belonging to genotypes G1 and G3 in cattle and sheep from four states in the USA as baseline data for future hypothesis-based studies. This is the first study to sequence *E. granulosus* from domestic ruminants in the USA. Sequences are available in GenBank (accession numbers: *cox1* sequences: OR398494-OR398496, *nad5* sequences: OR400695-OR400702).

In the domestic life cycle of *E. granulosus* in the USA, sheep, cattle and pigs have been previously shown to serve as intermediate hosts (Fig. 6) and hence potential sentinels of infections [7]. In abattoir studies, careful identification of cysts using molecular tests is essential to differentiate between Cysticercus tenuicollis—the metacestode of the non-zoonotic cestode *T. hydatigena*, and hydatid cyst—the metacestode of the zoonotic cestode *E. granulosus*. In this study, five out of 13 cysts isolated from sheep were identified as *T. hydatigena*. Identification and differentiation of *T. hydatigena* and *E. granulosus* can be performed accurately with molecular methods as demonstrated in this and prior studies [45, 46]. The presence of metacestodes of either species can lead to organ condemnation in abattoirs to prevent the entry of these parasites into human and animal food supplies, despite the metacestode stage not being directly infectious to humans.

Metacestodes of *E. granulosus* can be fertile or non-fertile [21]. Fertile metacestodes contain protoscolices, which when viable are infectious to definitive hosts (Fig. 1). Fertile protoscolices play a major role in perpetuating the life cycle. Non-fertile metacestodes do not contain protoscolices and are unable to infect definitive hosts. In this study, four out of eight hydatid cysts from sheep were fertile. However, all hydatid cysts from cattle in this study were non-fertile.

This is the first study to confirm the presence of genotypes G1 and G3 in domestic cattle and sheep in the USA. Genotypes G1-G3 belong to *E. granulosus* s.s. and are the most common genotypes reported globally. Previous reports from the USA have been restricted to *E. granulosus* s.l. genotypes G8 and G10, which are also referred to as *E. canadensis. E. canadensis* genotype G8 has been reported in humans [47] and genotypes G8 and G10 in dogs [48], wild canids [14, 16, 49] and wild cervids [50, 51]. In humans, *E. granulosus* G1-G3 causes the majority (88.4%) of cystic echinococcosis globally, followed by the G6/G7 genotypes of *E. canadensis* cluster (11.1%) and *E. ortleppi* (0.4%) [22].

We also identified for the first time, five unique *nad5* haplotypes and six unique *cox1-nad5* haplotypes of *E. granulosus* that have never been reported from any other part of the world (Figs. 3 and 5). These unique haplotypes appear to be endemic to the USA and were likely not introduced by the importation of infected animals. Two *nad5* haplotypes and one *cox1-nad5* haplotype identified in the study belonged to common global haplotypes. The novel *nad5* and *cox1-nad5* haplotypes identified within genotypes were supported by their positions in the median joining network (Fig. 3) and the Bayesian tree (Fig. 4). We have included country of origin in our *nad5* haplotypic nomenclature as used by Kinkar et al. [52]. However, there is a need to establish rules for haplotype nomenclature, but this may be hard since haplotypes identified in one gene (such as *nad5* in this study; Fig. 3) may not be identified in another gene (such as *cox1* in this study; Fig. 2).

Taken together, the molecular confirmation of infection in cattle and sheep indicates that there has been recent autochthonous *E. granulosus* transmission in the USA. Given that *E. granulosus* eggs can survive in the soil for up to a year (at 4 ℃ to 15 ℃) [53] and that detectable cysts can be seen in cattle and sheep within a year of infection [54], the presence of these cysts indicates pasture contamination and active infection in domestic and/or wild canids in the areas of origin of these infected animals within the last 5 years. Unless interventions are undertaken to identify, diagnose and treat definitive hosts which are the sources of the infection, transmission is likely to be ongoing. Deworming dogs with a broad-spectrum anthelmintic drug under the guidance of a licensed veterinarian and avoiding feeding dogs a raw food diet including restricting access to raw offal can help prevent zoonotic infections and reduce environmental contamination.

Further research is warranted to understand the epidemiology and risk factors associated with *Echinococcus* spp. in the USA. Surveillance for *E. granulosus* in dogs and wild canids living in association with domestic livestock and wild ungulates have been conducted to assess risk for *E. granulosus* s.l. spillover to humans in the 1960s in California [9], in the 1970s–1980s in Utah [11, 55, 56] and recently in Idaho [13], Maine [16], Minnesota [48], Montana [13] and Wyoming [14]. These, along with the present finding of hydatid cysts in sentinel intermediate hosts, highlight the need for further surveillance in canids at a national level. Current estimates of cystic echinococcosis prevalence in ruminant intermediate hosts in the USA are also unknown. Future studies are needed to establish the prevalence of infection in cattle, sheep and other domestic and wildlife intermediate hosts at a national level. Additionally, there is a need to establish a national public database of human cases and diagnostic guidelines to establish the genotype of infections. Molecular genotyping with haplotype analysis of cysts in infected humans must be encouraged to understand likely infection source/reservoir and to differentiate infections from *E. multilocularis*, another emerging parasitic infection reported in dogs and humans.

## Conclusions

In conclusion, we confirm the occurrence of *E. granulosus* s.s. genotypes G1 and G3 in cattle and sheep in the USA using nucleotide sequence data at two mitochondrial loci, *cox1* and *nad5*. G1 was the predominant genotype. Since domestic animals can be considered sentinels for echinococcosis, the presence of hydatid cysts in cattle and sheep indicates the likely presence of infected dogs and/or wild canids in sympatry at the areas of origin. Further molecular studies of prevalence in canids, intermediate hosts and humans are essential to understand the extent of infection in the USA. Understanding the occurrence of strains and species of *Echinococcus* will aid in understanding adverse risk factors for animal health, sustainability of meat production and potential sources of human disease.

### Supplementary Information

**Additional file1: Figure S1.** Multiple sequence alignment of the partial *nad5* sequences representing haplotypes in this study compared to the reference sequence AB786664 (nucleotides 727-1396). Twelve variable sites are colored. **Table S1.** Histological and molecular identification of samples analysed in this study. **Table S2.** Reports of autochthonous transmission of *Echinococcus granulosus* in the United States.

## Data Availability

Sequences generated in this study are available in the nucleotide database of GenBank under the following accession numbers: *cox1* sequences: OR398494-OR398496 and *nad5* sequences: OR400695-OR400702.
