# Supplementary material for: Cystic echinococcosis in cattle and sheep caused by Echinococcus granulosus sensu stricto genotypes G1 and G3 in the USA
Source: Parasit Vectors. 2024 Mar 14;17:128. doi: 10.1186/s13071-024-06192-x (PMC10938798; doi:10.1186/s13071-024-06192-x)
Supplement: Supplementary file 1 — Additional file1: Figure S1. Multiple sequence alignment of the partial nad5 sequences representing haplotypes in this study compared to the reference sequence AB786664 (nucleotides 727-1396). Twelve variable sites are colored. Table S1. Histological and molecular identification of samples analysed in this study. Table S2. Reports of autochthonous transmission of Echinococcus granulosus in the United States. [file 13071_2024_6192_MOESM1_ESM.docx]

**Additional Files:**


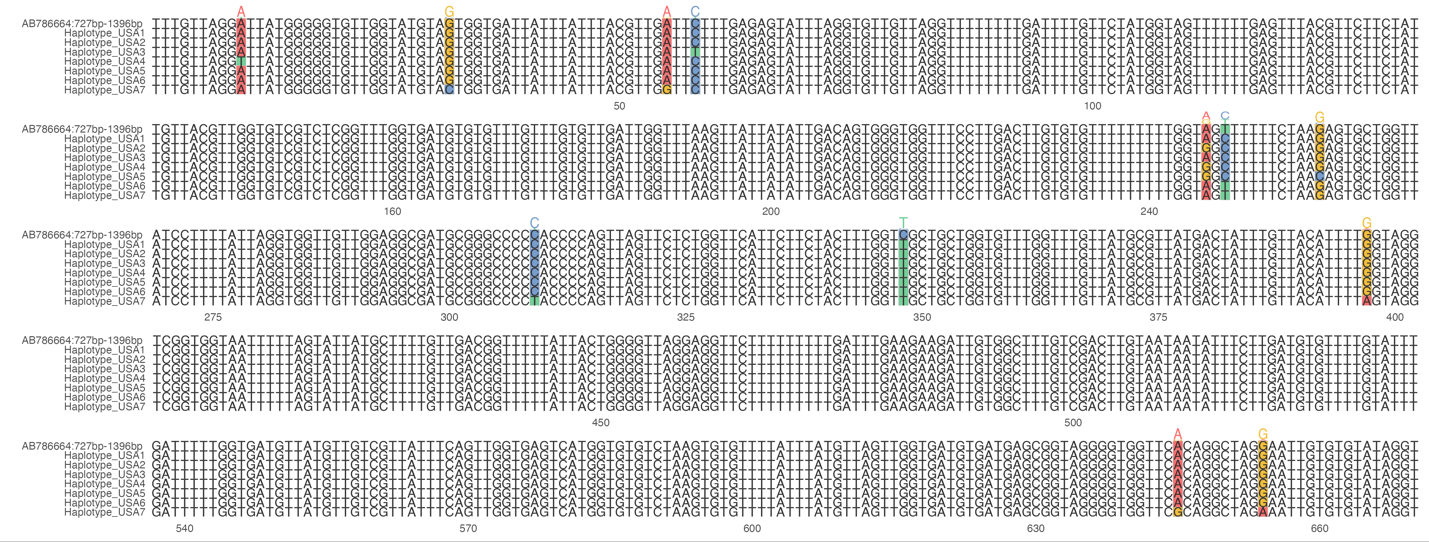


Figure S1. Multiple sequence alignment of the partial *nad5* sequences representing haplotypes in this study compared to the reference sequence AB786664 (nucleotides 727-1396). Twelve variable sites are colored.

Table S1. Histological and molecular identification of samples analysed in this study

| **Date** | **ILN** | **Abattoir state** | **Species** | **Histologic diagnosis** | **Fertility** | **Genotype** | **Genus species** | **nad5 haplotypes** |
| --- | --- | --- | --- | --- | --- | --- | --- | --- |
| 10/6/21 | B56948 | ID | Beef Cow | Hydatidosis | Non-fertile | G1 | *Echinococcus granulosus sensu stricto* | Haplotype USA 5 |
| 10/6/21 | B56949 | ID | Beef Cow | Hydatidosis | Non-fertile | G1 | *Echinococcus granulosus sensu stricto* | Haplotype USA 1 |
| 10/7/21 | B56970 | ID | Beef Cow | Hydatidosis | Non-fertile | G1 | *Echinococcus granulosus sensu stricto* | Haplotype USA 1 |
| 11/4/21 | B57262 | ID | Beef Cow | Hydatidosis | Non-fertile | G1 | *Echinococcus granulosus sensu stricto* | Haplotype USA 1 |
| 11/9/21 | B57305 | ID | Beef Cow | Hydatidosis | Non-fertile | G1 | *Echinococcus granulosus sensu stricto* | Haplotype USA 1 |
| 11/9/21 | B57306 | ID | Beef Cow | Degenerate cyst (likely hydatidosis) | Non-fertile | G1 | *Echinococcus granulosus sensu stricto* | Haplotype USA 1 |
| 11/12/21 | B57351 | ID | Beef Cow | Hydatidosis | Non-fertile | G1 | *Echinococcus granulosus sensu stricto* | Haplotype USA 1 |
| 11/29/21 | B57469 | ID | Beef Cow | Hydatidosis | Non-fertile | G1 | *Echinococcus granulosus sensu stricto* | Haplotype USA 1 |
| 11/29/21 | B57470 | ID | Beef Cow | Hydatidosis | Non-fertile | G1 | *Echinococcus granulosus sensu stricto* | Haplotype USA 1 |
| 11/29/21 | B57471 | ID | Beef Cow | Hydatidosis | Non-fertile | G1 | *Echinococcus granulosus sensu stricto* | Haplotype USA 1 |
| 12/6/21 | B57523 | ID | Beef Cow | Hydatidosis | Non-fertile | G1 | *Echinococcus granulosus sensu stricto* | Haplotype USA 1 |
| 12/6/21 | B57524 | ID | Beef Cow | Hydatidosis | Non-fertile | G1 | *Echinococcus granulosus sensu stricto* | Haplotype USA 1 |
| 12/6/21 | B57525 | ID | Beef Cow | Hydatidosis | Non-fertile | G1 | *Echinococcus granulosus sensu stricto* | Haplotype USA 1 |
| 12/6/21 | B57526 | ID | Beef Cow | Hydatidosis | Non-fertile | G1 | *Echinococcus granulosus sensu stricto* | Haplotype USA 1 |
| 12/6/21 | B57527 | ID | Beef Cow | Hydatidosis | Non-fertile | G1 | *Echinococcus granulosus sensu stricto* | Haplotype USA 1 |
| 12/6/21 | B57528 | ID | Beef Cow | Hydatidosis | Non-fertile | G1 | *Echinococcus granulosus sensu stricto* | Haplotype USA 1 |
| 12/6/21 | B57529 | ID | Beef Cow | Hydatidosis | Non-fertile | G1 | *Echinococcus granulosus sensu stricto* | Haplotype USA 1 |
| 12/6/21 | B57534 | ID | Beef Cow | Hydatidosis | Non-fertile | G1 | *Echinococcus granulosus sensu stricto* | Haplotype USA 1 |
| 12/6/21 | B57535 | ID | Beef Cow | Hydatidosis | Non-fertile | G1 | *Echinococcus granulosus sensu stricto* | Haplotype USA 1 |
| 12/6/21 | B57536 | ID | Beef Cow | Hydatidosis | Non-fertile | G1 | *Echinococcus granulosus sensu stricto* | Haplotype USA 1 |
| 12/10/21 | B57599 | ID | Beef Cow | Hydatidosis | Non-fertile | G1 | *Echinococcus granulosus sensu stricto* | Haplotype USA 1 |
| 12/20/21 | B57656 | NY | Mature Sheep | Cysticercus tenuicollis |  | -- | *Taenia hydatigena* |  |
| 12/20/21 | B57659 | ID | Beef Cow | Hydatidosis | Non-fertile | G1 | *Echinococcus granulosus sensu stricto* | Haplotype USA 2 |
| 12/22/21 | B57682 | ID | Beef Cow | Hydatidosis | Non-fertile | G1 | *Echinococcus granulosus sensu stricto* | Haplotype USA 1 |
| 12/22/21 | B57683 | ID | Beef Cow | Hydatidosis | Non-fertile | G1 | *Echinococcus granulosus sensu stricto* | Haplotype USA 2 |
| 12/27/21 | B57705 | ID | Beef Cow | Hydatidosis | Non-fertile | G1 | *Echinococcus granulosus sensu stricto* | Haplotype USA 1 |
| 12/27/21 | B57706 | ID | Beef Cow | Hydatidosis | Non-fertile | G1 | *Echinococcus granulosus sensu stricto* | Haplotype USA 1 |
| 12/29/21 | B57732 | NY | Lamb | Hydatidosis | **Fertile** | G1 | *Echinococcus granulosus sensu stricto* | Haplotype USA 1 |
| 12/30/21 | B57737 | PA | Mature Sheep | Bacterial pyogranuloma | - | G1 | *Echinococcus granulosus sensu stricto* | - |
| 12/30/21 | B57738 | PA | Mature Sheep | Bacterial pyogranuloma | - | G1 | *Echinococcus granulosus sensu stricto* | - |
| 12/30/21 | B57739 | PA | Mature Sheep | Hydatidosis | Non-fertile | G1 | *Echinococcus granulosus sensu stricto* | Haplotype USA 1 |
| 1/3/22 | B57760 | ID | Beef Cow | Bacterial abscess | - | G1 | *Echinococcus granulosus sensu stricto* | - |
| 1/10/22 | B57812 | ID | Beef Cow | Hydatidosis | Non-fertile | G1 | *Echinococcus granulosus sensu stricto* | Haplotype USA 1 |
| 1/10/22 | B57813 | ID | Beef Cow | Hydatidosis | Non-fertile | G1 | *Echinococcus granulosus sensu stricto* | Haplotype USA 1 |
| 1/12/22 | B57841 | ID | Beef Cow | Hydatidosis | Non-fertile | G1 | *Echinococcus granulosus sensu stricto* | Haplotype USA 2 |
| 1/12/22 | B57842 | ID | Beef Cow | Hydatidosis | Non-fertile | G1 | *Echinococcus granulosus sensu stricto* | Haplotype USA 1 |
| 1/12/22 | B57843 | ID | Beef Cow | Hydatidosis | Non-fertile | G1 | *Echinococcus granulosus sensu stricto* | Haplotype USA 1 |
| 1/14/22 | B57887 | ID | Beef Cow | Hydatidosis | Non-fertile | G1 | *Echinococcus granulosus sensu stricto* | Haplotype USA 2 |
| 1/19/22 | B57915 | ID | Beef Cow | Hydatidosis | Non-fertile | G1 | *Echinococcus granulosus sensu stricto* | Haplotype USA 1 |
| 1/19/22 | B57916 | ID | Beef Cow | Hydatidosis | Non-fertile | G1 | *Echinococcus granulosus sensu stricto* | Haplotype USA 2 |
| 1/19/22 | B57917 | ID | Beef Cow | Degenerate cyst (likely hydatidosis) | - | G1 | *Echinococcus granulosus sensu stricto* | Haplotype USA 2 |
| 1/19/22 | B57918 | ID | Beef Cow | Hydatidosis | Non-fertile | G1 | *Echinococcus granulosus sensu stricto* | Haplotype USA 2 |
| 1/20/22 | B57927 | ID | Beef Cow | Degenerate cyst (likely hydatidosis) | - | G1 | *Echinococcus granulosus sensu stricto* | Haplotype USA 4 |
| 1/20/22 | B57928 | ID | Beef Cow | Pulmonary atelectasis (no cyst) | - | G1 | *Echinococcus granulosus sensu stricto* | Haplotype USA 2 |
| 1/20/22 | B57929 | ID | Dairy Cow | Hydatidosis | Non-fertile | G1 | *Echinococcus granulosus sensu stricto* | Haplotype USA 1 |
| 1/25/22 | B57965 | ID | Dairy Cow | Hydatidosis | Non-fertile | G1 | *Echinococcus granulosus sensu stricto* | Haplotype USA 2 |
| 1/27/22 | B58002 | ID | Beef Cow | Hydatidosis | Non-fertile | G1 | *Echinococcus granulosus sensu stricto* | Haplotype USA6 |
| 1/28/22 | B58006 | ID | Beef Cow | Hydatidosis | Non-fertile | G1 | *Echinococcus granulosus sensu stricto* | Haplotype USA 1 |
| 1/28/22 | B58007 | ID | Beef Cow | Hydatidosis | Non-fertile | G3 | *Echinococcus granulosus sensu stricto* | Haplotype USA 7 |
| 1/28/22 | B58008 | ID | Beef Cow | Hydatidosis | Non-fertile | G3 | *Echinococcus granulosus sensu stricto* | Haplotype USA 7 |
| 2/7/22 | B58107 | NY | Mature Sheep | Hydatidosis | **Fertile** | G1 | *Echinococcus granulosus sensu stricto* | Haplotype USA 1 |
| 2/7/22 | B58108 | NY | Mature Sheep | Hydatidosis | Non-fertile | G1 | *Echinococcus granulosus sensu stricto* | Haplotype USA 1 |
| 2/7/22 | B58109 | NY | Mature Sheep | Hydatidosis | **Fertile** | G1 | *Echinococcus granulosus sensu stricto* | Haplotype USA 1 |
| 2/9/22 | B58135 | ID | Beef Cow | Hydatidosis | Non-fertile | G1 | *Echinococcus granulosus sensu stricto* | Haplotype USA 1 |
| 2/9/22 | B58136 | ID | Beef Cow | Hydatidosis | Non-fertile | G1 | *Echinococcus granulosus sensu stricto* | Haplotype USA 2 |
| 2/9/22 | B58142 | ID | Beef Cow | Hydatidosis | Non-fertile | G1 | *Echinococcus granulosus sensu stricto* | Haplotype USA 2 |
| 2/10/22 | B58163 | ID | Beef Cow | Hydatidosis | Non-fertile | G1 | *Echinococcus granulosus sensu stricto* | Haplotype USA 3 |
| 2/11/22 | B58180 | ID | Beef Cow | Hydatidosis | Non-fertile | G1 | *Echinococcus granulosus sensu stricto* | Haplotype USA 2 |
| 2/11/22 | B58181 | ID | Beef Cow | Hydatidosis | Non-fertile | G1 | *Echinococcus granulosus sensu stricto* | Haplotype USA 2 |
| 2/16/22 | B58238 | ID | Beef Cow | Hydatidosis | Non-fertile | G1 | *Echinococcus granulosus sensu stricto* | Haplotype USA 2 |
| 2/22/22 | B58279 | ID | Dairy Cow | Hydatidosis | Non-fertile | G1 | *Echinococcus granulosus sensu stricto* | Haplotype USA 1 |
| 3/1/22 | B58381 | NY | Mature Sheep | Hydatidosis | **Fertile** | G1 | *Echinococcus granulosus sensu stricto* | Haplotype USA 1 |
| 3/8/22 | B58452 | ID | Beef Cow | Hydatidosis | Non-fertile | G1 | *Echinococcus granulosus sensu stricto* | Haplotype USA 2 |
| 3/8/22 | B58471 | ID | Dairy Cow | Bile duct cyst | Non-fertile | G1 | *Echinococcus granulosus sensu stricto* | Haplotype USA 1 |
| 3/9/22 | B58491 | NY | Mature Sheep | Cysticercus tenuicollis | - | - | *Taenia hydatigena* | - |
| 3/21/22 | B58589 | ID | Beef Cow | Hydatidosis | Non-fertile | G1 | *Echinococcus granulosus sensu stricto* | Haplotype USA 1 |
| 3/23/22 | B58621 | ID | Beef Cow | Hydatidosis | Non-fertile | G1 | *Echinococcus granulosus sensu stricto* | Haplotype USA 1 |
| 4/8/22 | B58810 | ID | Beef Cow | Hydatidosis | Non-fertile | G1 | *Echinococcus granulosus sensu stricto* | Haplotype USA 1 |
| 4/11/22 | B58823 | ID | Beef Cow | Hydatidosis | Non-fertile | G1 | *Echinococcus granulosus sensu stricto* | Haplotype USA 1 |
| 4/14/22 | B58860 | ID | Beef Cow | Hydatidosis | Non-fertile | G1 | *Echinococcus granulosus sensu stricto* | Haplotype USA 2 |
| 4/20/22 | B58910 | ID | Dairy Cow | Pneumonia (no cyst) | - | G1 | *Echinococcus granulosus sensu stricto* | Haplotype USA 1 |
| 5/26/22 | B59244 | ID | Beef Cow | Hydatidosis | Non-fertile | G3 | *Echinococcus granulosus sensu stricto* | Haplotype USA 7 |
| 6/3/22 | B59301 | ID | Beef Cow | Hydatidosis | Non-fertile | G1 | *Echinococcus granulosus sensu stricto* | Haplotype USA 1 |
| 6/3/22 | B59302 | ID | Beef Cow | Hydatidosis | Non-fertile | G1 | *Echinococcus granulosus sensu stricto* | Haplotype USA 1 |
| 6/7/22 | B59331 | ID | Beef Cow | Hydatidosis | Non-fertile | G1 | *Echinococcus granulosus sensu stricto* | Haplotype USA 1 |
| 6/17/22 | B59412 | ID | Beef Cow | Hydatidosis | Non-fertile | G1 | *Echinococcus granulosus sensu stricto* | Haplotype USA 1 |
| 6/21/22 | B59427 | ID | Beef Cow | Hydatidosis | Non-fertile | G1 | *Echinococcus granulosus sensu stricto* | Haplotype USA 1 |
| 6/22/22 | B59449 | WI | Lamb | Cysticercus tenuicollis | - | -- | *Taenia hydatigena* | - |
| 6/24/22 | B59474 | ID | Beef Cow | Hydatidosis | Non-fertile | G1 | *Echinococcus granulosus sensu stricto* | Haplotype USA 2 |
| 6/24/22 | B59475 | ID | Beef Cow | Hydatidosis | Non-fertile | G1 | *Echinococcus granulosus sensu stricto* | Haplotype USA 1 |
| 6/24/22 | B59476 | ID | Beef Cow | Hydatidosis | Non-fertile | G1 | *Echinococcus granulosus sensu stricto* | Haplotype USA 1 |
| 6/30/22 | B59520 | ID | Beef Cow | Hydatidosis | Non-fertile | G1 | *Echinococcus granulosus sensu stricto* | Haplotype USA 1 |
| 7/8/22 | B59569 | ID | Beef Cow | Hydatidosis | Non-fertile | G1 | *Echinococcus granulosus sensu stricto* | Haplotype USA 1 |
| 7/8/22 | B59570 | ID | Beef Cow | Hydatidosis | Non-fertile | G1 | *Echinococcus granulosus sensu stricto* | Haplotype USA 1 |
| 7/8/22 | B59571 | ID | Beef Cow | Hydatidosis | Non-fertile | G1 | *Echinococcus granulosus sensu stricto* | Haplotype USA 1 |
| 7/13/22 | B59610 | WI | Lamb | Cysticercus tenuicollis | - | -- | *Taenia hydatigena* | - |
| 7/20/22 | B59667 | WI | Lamb | Cysticercus tenuicollis | - | -- | *Taenia hydatigena* | - |
| 8/1/22 | B59764 | ID | Beef Cow | Hydatidosis | Non-fertile | G1 | *Echinococcus granulosus sensu stricto* | Haplotype USA 1 |
| 8/22/22 | B59908 | ID | Beef Cow | Hydatidosis | Non-fertile | G1 | *Echinococcus granulosus sensu stricto* | Haplotype USA 1 |
| 8/31/22 | B59978 | ID | Beef Cow | Hydatidosis | Non-fertile | G1 | *Echinococcus granulosus sensu stricto* | Haplotype USA 1 |
| 9/13/22 | B60068 | ID | Beef Cow | Hydatidosis | Non-fertile | G1 | *Echinococcus granulosus sensu stricto* | Haplotype USA 1 |
| 10/13/22 | B60300 | ID | Beef Cow | Degenerate cyst (likely hydatidosis) | - | G1 | *Echinococcus granulosus sensu stricto* | Haplotype USA 1 |
| 10/13/22 | B60301 | ID | Beef Cow | Hydatidosis | Non-fertile | G1 | *Echinococcus granulosus sensu stricto* | Haplotype USA 1 |
| 10/14/22 | B60317 | ID | Beef Cow | Hydatidosis | Non-fertile | G1 | *Echinococcus granulosus sensu stricto* | Haplotype USA 1 |

Table S2. Reports of autochthonous transmission of *Echinococcus granulosus* in the United States.

| U.S. State | Years | Humans | Dogs | Sheep | Pigs/cattle | Deer/Moose/elk | Wild canids | References |
| --- | --- | --- | --- | --- | --- | --- | --- | --- |
| Alabama | 1941-1950 |  |  |  |  |  |  |  |
| Alabama | 1951-1960 |  |  |  |  |  |  |  |
| Alabama | 1961-1970 |  |  |  |  |  |  |  |
| Alabama | 1971-1980 |  |  |  |  |  |  |  |
| Alabama | 1800-1940 |  |  |  |  |  |  |  |
| Alabama | 1980-2000 |  |  |  |  |  |  |  |
| Alabama | 2000-2023 |  |  |  |  |  |  |  |
| Alaska | 1941-1950 | Reported | Reported |  |  | Reported | Reported | [1] |
| Alaska | 1951-1960 | Reported | Reported |  |  | Reported | Reported | [1] |
| Alaska | 1961-1970 | Reported |  |  |  |  |  | [2] |
| Alaska | 1971-1980 | Reported |  |  |  |  |  | [3] |
| Alaska | 1800-1940 |  | Reported |  |  | Reported |  | [4, 5] |
| Alaska | 1980-2000 | Reported |  |  |  |  |  | [6] |
| Alaska | 2000-2023 | Reported |  |  |  | Reported |  | [7, 8] |
| Arizona | 1941-1950 |  |  |  |  |  |  |  |
| Arizona | 1951-1960 |  |  |  |  |  |  |  |
| Arizona | 1961-1970 | Reported |  |  |  |  |  | [9] |
| Arizona | 1971-1980 | Reported | Reported |  |  |  |  | [10, 11] |
| Arizona | 1800-1940 |  |  |  |  |  |  |  |
| Arizona | 1980-2000 |  |  |  |  |  |  |  |
| Arizona | 2000-2023 |  |  |  |  |  |  |  |
| Arkansas | 1941-1950 |  |  |  | Reported |  |  | [12] |
| Arkansas | 1951-1960 | Reported |  |  |  |  |  | [13, 14] |
| Arkansas | 1961-1970 |  |  |  |  |  |  |  |
| Arkansas | 1971-1980 |  |  |  |  |  |  |  |
| Arkansas | 1800-1940 | Reported |  |  |  |  |  | [9, 15] |
| Arkansas | 1980-2000 |  |  |  |  |  |  |  |
| Arkansas | 2000-2023 |  |  |  |  |  |  |  |
| California | 1941-1950 | Reported |  |  |  | Reported |  | [16, 17] |
| California | 1951-1960 | Reported | Reported | Reported |  | Reported |  | [16, 18] |
| California | 1961-1970 | Reported | Reported | Reported |  | Reported | Reported | [16, 18, 19, 20, 21, 22] |
| California | 1971-1980 |  |  |  |  |  |  |  |
| California | 1800-1940 | Reported |  |  |  | Reported | Reported | [5, 23, 24] |
| California | 1980-2000 |  |  |  |  |  |  |  |
| California | 2000-2023 | Reported |  |  |  |  |  | [25] |
| Colorado | 1941-1950 |  |  |  |  |  |  |  |
| Colorado | 1951-1960 |  |  |  |  |  |  |  |
| Colorado | 1961-1970 |  |  |  |  |  |  |  |
| Colorado | 1971-1980 |  |  |  |  |  |  |  |
| Colorado | 1800-1940 |  |  |  |  |  |  |  |
| Colorado | 1980-2000 |  |  |  |  |  |  |  |
| Colorado | 2000-2023 |  |  |  |  |  |  |  |
| Connecticut | 1941-1950 |  |  |  |  |  |  |  |
| Connecticut | 1951-1960 |  |  |  |  |  |  |  |
| Connecticut | 1961-1970 |  |  |  |  |  |  |  |
| Connecticut | 1971-1980 |  |  |  |  |  |  |  |
| Connecticut | 1800-1940 |  |  |  |  |  |  |  |
| Connecticut | 1980-2000 |  |  |  |  |  |  |  |
| Connecticut | 2000-2023 |  |  |  |  |  |  |  |
| Delaware | 1941-1950 |  |  |  |  |  |  |  |
| Delaware | 1951-1960 |  |  |  |  |  |  |  |
| Delaware | 1961-1970 |  |  |  |  |  |  |  |
| Delaware | 1971-1980 |  |  |  |  |  |  |  |
| Delaware | 1800-1940 |  |  |  |  |  |  |  |
| Delaware | 1980-2000 |  |  |  |  |  |  |  |
| Delaware | 2000-2023 |  |  |  |  |  |  |  |
| Florida | 1941-1950 | Reported |  |  |  |  |  | [26] |
| Florida | 1951-1960 |  |  |  |  |  |  |  |
| Florida | 1961-1970 |  |  |  |  |  |  |  |
| Florida | 1971-1980 |  |  |  |  |  |  |  |
| Florida | 1800-1940 |  |  |  |  |  |  |  |
| Florida | 1980-2000 |  |  |  |  |  |  |  |
| Florida | 2000-2023 |  |  |  |  |  |  |  |
| Georgia | 1941-1950 |  |  |  |  |  |  |  |
| Georgia | 1951-1960 | Reported |  |  |  |  |  | [14] |
| Georgia | 1961-1970 |  |  |  |  |  |  |  |
| Georgia | 1971-1980 |  |  |  |  |  |  |  |
| Georgia | 1800-1940 |  | Reported |  |  |  |  | [5, 13] |
| Georgia | 1980-2000 |  |  |  |  |  |  |  |
| Georgia | 2000-2023 |  |  |  |  |  |  |  |
| Hawaii | 1941-1950 |  |  |  |  |  |  |  |
| Hawaii | 1951-1960 |  |  |  |  |  |  |  |
| Hawaii | 1961-1970 |  |  |  |  |  |  |  |
| Hawaii | 1971-1980 |  |  |  |  |  |  |  |
| Hawaii | 1800-1940 |  |  |  |  |  |  |  |
| Hawaii | 1980-2000 |  |  |  |  |  |  |  |
| Hawaii | 2000-2023 |  |  |  |  |  |  |  |
| Idaho | 1941-1950 | Reported |  |  |  |  |  | [27] |
| Idaho | 1951-1960 |  |  |  |  |  |  |  |
| Idaho | 1961-1970 |  |  |  |  |  |  |  |
| Idaho | 1971-1980 |  |  |  |  |  |  |  |
| Idaho | 1800-1940 |  |  |  |  |  |  |  |
| Idaho | 1980-2000 |  |  |  |  |  |  |  |
| Idaho | 2000-2023 |  |  |  |  | Reported | Reported | [28, 29] |
| Illinois | 1941-1950 |  |  |  |  |  |  |  |
| Illinois | 1951-1960 |  |  |  |  |  |  |  |
| Illinois | 1961-1970 |  |  |  |  |  |  |  |
| Illinois | 1971-1980 |  |  |  |  |  |  |  |
| Illinois | 1800-1940 | Reported |  |  |  |  |  | [9] |
| Illinois | 1980-2000 |  |  |  |  |  |  |  |
| Illinois | 2000-2023 |  |  |  |  |  |  |  |
| Indiana | 1941-1950 |  |  |  |  |  |  |  |
| Indiana | 1951-1960 | Reported |  |  |  |  |  | [14] |
| Indiana | 1961-1970 |  |  |  |  |  |  |  |
| Indiana | 1971-1980 |  |  |  |  |  |  |  |
| Indiana | 1800-1940 | Reported |  |  | Reported |  |  | [9, 30] |
| Indiana | 1980-2000 |  |  |  |  |  |  |  |
| Indiana | 2000-2023 |  |  |  |  |  |  |  |
| Iowa | 1941-1950 |  |  |  |  |  |  |  |
| Iowa | 1951-1960 |  |  |  |  |  |  |  |
| Iowa | 1961-1970 |  |  |  |  |  |  |  |
| Iowa | 1971-1980 |  |  |  |  |  |  |  |
| Iowa | 1800-1940 |  |  |  | Reported |  |  | [30] |
| Iowa | 1980-2000 |  |  |  |  |  |  |  |
| Iowa | 2000-2023 |  |  |  |  |  |  |  |
| Kansas | 1941-1950 |  |  |  |  |  |  |  |
| Kansas | 1951-1960 |  |  |  |  |  |  |  |
| Kansas | 1961-1970 |  |  |  |  |  |  |  |
| Kansas | 1971-1980 |  |  |  |  |  |  |  |
| Kansas | 1800-1940 |  |  |  |  |  |  |  |
| Kansas | 1980-2000 |  |  |  |  |  |  |  |
| Kansas | 2000-2023 |  |  |  |  |  |  |  |
| Kentucky | 1941-1950 |  | Reported |  |  |  |  | [5, 13] |
| Kentucky | 1951-1960 |  |  |  |  |  |  |  |
| Kentucky | 1961-1970 |  |  |  |  |  |  |  |
| Kentucky | 1971-1980 |  |  |  |  |  |  |  |
| Kentucky | 1800-1940 |  |  |  | Reported |  |  | [30] |
| Kentucky | 1980-2000 |  |  |  |  |  |  |  |
| Kentucky | 2000-2023 |  |  |  |  |  |  |  |
| Louisiana | 1941-1950 | Reported |  |  |  |  |  | [31] |
| Louisiana | 1951-1960 | Reported |  |  |  |  |  | [13] |
| Louisiana | 1961-1970 |  |  |  |  |  |  |  |
| Louisiana | 1971-1980 |  |  |  |  |  |  |  |
| Louisiana | 1800-1940 | Reported |  |  | Reported |  |  | [32] |
| Louisiana | 1980-2000 |  |  |  |  |  |  |  |
| Louisiana | 2000-2023 |  |  |  |  |  |  |  |
| Maine | 1941-1950 |  |  |  |  |  |  |  |
| Maine | 1951-1960 |  |  |  |  |  |  |  |
| Maine | 1961-1970 |  |  |  |  |  |  |  |
| Maine | 1971-1980 |  |  |  |  |  |  |  |
| Maine | 1800-1940 |  |  |  |  |  |  |  |
| Maine | 1980-2000 |  |  |  |  |  |  |  |
| Maine | 2000-2023 |  |  |  |  | Reported | Reported | [33, 34] |
| Maryland | 1941-1950 |  |  |  |  |  |  |  |
| Maryland | 1951-1960 |  |  |  |  |  |  |  |
| Maryland | 1961-1970 |  |  |  |  |  |  |  |
| Maryland | 1971-1980 |  |  |  |  |  |  |  |
| Maryland | 1800-1940 |  |  |  |  |  |  |  |
| Maryland | 1980-2000 |  |  |  |  |  |  |  |
| Maryland | 2000-2023 |  |  |  |  |  |  |  |
| Massachusetts | 1941-1950 | Reported |  |  |  |  |  | [14] |
| Massachusetts | 1951-1960 |  |  |  |  |  |  |  |
| Massachusetts | 1961-1970 |  |  |  |  |  |  |  |
| Massachusetts | 1971-1980 |  |  |  |  |  |  |  |
| Massachusetts | 1800-1940 |  |  |  |  |  |  |  |
| Massachusetts | 1980-2000 |  |  |  |  |  |  |  |
| Massachusetts | 2000-2023 |  |  |  |  |  |  |  |
| Michigan | 1941-1950 |  |  |  |  |  |  |  |
| Michigan | 1951-1960 |  |  |  |  |  |  |  |
| Michigan | 1961-1970 |  |  |  |  |  |  |  |
| Michigan | 1971-1980 |  |  |  |  |  |  |  |
| Michigan | 1800-1940 |  |  |  |  | Reported |  | [5] |
| Michigan | 1980-2000 |  |  |  |  |  |  |  |
| Michigan | 2000-2023 |  |  |  |  |  |  |  |
| Minnesota | 1941-1950 |  |  |  |  | Reported | Reported | [35, 36] |
| Minnesota | 1951-1960 | Reported |  |  |  |  | Reported | [14, 36] |
| Minnesota | 1961-1970 |  |  |  |  |  |  |  |
| Minnesota | 1971-1980 |  |  |  |  |  |  |  |
| Minnesota | 1800-1940 | Reported |  |  |  | Reported | Reported | [4, 5, 9, 35] |
| Minnesota | 1980-2000 |  |  |  |  |  |  |  |
| Minnesota | 2000-2023 |  | Reported |  |  |  | Reported | [37] |
| Mississippi | 1941-1950 |  |  |  |  |  |  |  |
| Mississippi | 1951-1960 | Reported | Reported |  | Reported |  |  | [5, 13, 38, 39, 40, 41] |
| Mississippi | 1961-1970 | Reported | Reported |  |  |  |  | [18, 41] |
| Mississippi | 1971-1980 |  |  |  |  |  |  |  |
| Mississippi | 1800-1940 | Reported |  |  |  |  |  | [40] |
| Mississippi | 1980-2000 | Reported |  |  |  |  |  | [42] |
| Mississippi | 2000-2023 |  |  |  |  |  |  |  |
| Missouri | 1941-1950 |  |  |  |  |  |  |  |
| Missouri | 1951-1960 | Reported |  |  |  |  |  | [14] |
| Missouri | 1961-1970 |  |  |  |  |  |  |  |
| Missouri | 1971-1980 |  |  |  |  |  |  |  |
| Missouri | 1800-1940 | Reported |  |  | Reported |  |  | [9, 30] |
| Missouri | 1980-2000 |  |  |  |  |  |  |  |
| Missouri | 2000-2023 |  |  |  |  |  |  |  |
| Montana | 1941-1950 |  |  |  |  |  |  |  |
| Montana | 1951-1960 |  |  |  |  |  |  |  |
| Montana | 1961-1970 |  |  |  |  |  |  |  |
| Montana | 1971-1980 |  |  |  |  | Reported |  | [43] |
| Montana | 1800-1940 |  |  |  |  |  |  |  |
| Montana | 1980-2000 |  |  |  |  |  |  |  |
| Montana | 2000-2023 |  |  |  |  | Reported | Reported | [29] |
| Nebraska | 1941-1950 |  |  |  |  |  |  |  |
| Nebraska | 1951-1960 |  |  |  |  |  |  |  |
| Nebraska | 1961-1970 |  |  |  |  |  |  |  |
| Nebraska | 1971-1980 |  |  |  |  |  |  |  |
| Nebraska | 1800-1940 |  |  |  |  |  |  |  |
| Nebraska | 1980-2000 |  |  |  |  |  |  |  |
| Nebraska | 2000-2023 |  |  |  |  |  |  |  |
| Nevada | 1941-1950 | Reported |  |  |  |  |  | [9] |
| Nevada | 1951-1960 |  |  |  |  |  |  |  |
| Nevada | 1961-1970 | Reported |  |  |  |  |  | [9] |
| Nevada | 1971-1980 | Reported |  |  |  |  |  | [9] |
| Nevada | 1800-1940 |  |  |  |  |  |  |  |
| Nevada | 1980-2000 |  |  |  |  |  |  |  |
| Nevada | 2000-2023 |  |  |  |  |  |  |  |
| New Hampshire | 1941-1950 |  |  |  |  |  |  |  |
| New Hampshire | 1951-1960 |  |  |  |  |  |  |  |
| New Hampshire | 1961-1970 |  |  |  |  |  |  |  |
| New Hampshire | 1971-1980 |  |  |  |  |  |  |  |
| New Hampshire | 1800-1940 |  |  |  |  |  |  |  |
| New Hampshire | 1980-2000 |  |  |  |  |  |  |  |
| New Hampshire | 2000-2023 | Reported |  |  |  |  |  | [44] |
| New Jersey | 1941-1950 |  |  |  |  |  |  |  |
| New Jersey | 1951-1960 |  |  |  |  |  |  |  |
| New Jersey | 1961-1970 |  |  |  |  |  |  |  |
| New Jersey | 1971-1980 |  |  |  |  |  |  |  |
| New Jersey | 1800-1940 |  |  |  |  |  |  |  |
| New Jersey | 1980-2000 |  |  |  |  |  |  |  |
| New Jersey | 2000-2023 |  |  |  |  |  |  |  |
| New Mexico | 1941-1950 |  |  |  |  |  |  |  |
| New Mexico | 1951-1960 |  |  |  |  |  |  |  |
| New Mexico | 1961-1970 | Reported |  |  |  |  |  | [45] |
| New Mexico | 1971-1980 | Reported | Reported |  |  |  |  | [10, 11] |
| New Mexico | 1800-1940 |  |  |  |  |  |  |  |
| New Mexico | 1980-2000 |  |  |  |  |  |  |  |
| New Mexico | 2000-2023 |  |  |  |  |  |  |  |
| New York | 1941-1950 |  |  |  |  |  |  |  |
| New York | 1951-1960 | Reported |  |  |  |  |  | [14] |
| New York | 1961-1970 |  |  |  |  |  |  |  |
| New York | 1971-1980 |  |  |  |  |  |  |  |
| New York | 1800-1940 | Reported |  |  |  |  |  | [9] |
| New York | 1980-2000 |  |  |  |  |  |  |  |
| New York | 2000-2023 |  |  |  |  |  |  |  |
| North Carolina | 1941-1950 |  |  |  |  |  |  |  |
| North Carolina | 1951-1960 |  |  |  |  |  |  |  |
| North Carolina | 1961-1970 |  |  |  |  |  |  |  |
| North Carolina | 1971-1980 |  |  |  |  |  |  |  |
| North Carolina | 1800-1940 |  |  |  |  |  |  |  |
| North Carolina | 1980-2000 |  |  |  |  |  |  |  |
| North Carolina | 2000-2023 |  |  |  |  |  |  |  |
| North Dakota | 1941-1950 |  |  |  |  |  |  |  |
| North Dakota | 1951-1960 |  |  |  |  |  |  |  |
| North Dakota | 1961-1970 |  |  |  |  |  |  |  |
| North Dakota | 1971-1980 |  |  |  |  |  |  |  |
| North Dakota | 1800-1940 |  |  |  |  |  |  |  |
| North Dakota | 1980-2000 |  |  |  |  |  |  |  |
| North Dakota | 2000-2023 |  |  |  |  |  |  |  |
| Ohio | 1941-1950 |  |  |  |  |  |  |  |
| Ohio | 1951-1960 |  |  |  |  |  |  |  |
| Ohio | 1961-1970 |  |  |  |  |  |  |  |
| Ohio | 1971-1980 |  |  |  |  |  |  |  |
| Ohio | 1800-1940 |  |  |  | Reported |  |  | [32] |
| Ohio | 1980-2000 |  |  |  |  |  |  |  |
| Ohio | 2000-2023 |  |  |  |  |  |  |  |
| Oklahoma | 1941-1950 |  |  |  | Reported |  |  | [12] |
| Oklahoma | 1951-1960 |  |  |  |  |  |  |  |
| Oklahoma | 1961-1970 |  |  |  |  |  |  |  |
| Oklahoma | 1971-1980 |  |  |  |  |  |  |  |
| Oklahoma | 1800-1940 |  |  |  |  |  |  |  |
| Oklahoma | 1980-2000 |  |  |  |  |  |  |  |
| Oklahoma | 2000-2023 |  |  |  |  |  |  |  |
| Oregon | 1941-1950 |  |  |  |  |  |  |  |
| Oregon | 1951-1960 |  |  |  |  |  |  |  |
| Oregon | 1961-1970 | Reported |  |  |  |  |  | [9] |
| Oregon | 1971-1980 |  |  |  |  |  |  |  |
| Oregon | 1800-1940 |  |  |  |  | Reported |  | [46] |
| Oregon | 1980-2000 |  |  |  |  |  |  |  |
| Oregon | 2000-2023 |  |  |  |  |  |  |  |
| Pennsylvania | 1941-1950 |  |  |  |  |  |  |  |
| Pennsylvania | 1951-1960 |  |  |  |  |  |  |  |
| Pennsylvania | 1961-1970 |  |  |  |  |  |  |  |
| Pennsylvania | 1971-1980 |  |  |  |  |  |  |  |
| Pennsylvania | 1800-1940 |  |  |  |  | Reported |  | [5] |
| Pennsylvania | 1980-2000 |  |  |  |  |  |  |  |
| Pennsylvania | 2000-2023 |  |  |  |  |  | Reported | [44] |
| Rhode Island | 1941-1950 |  |  |  |  |  |  |  |
| Rhode Island | 1951-1960 |  |  |  |  |  |  |  |
| Rhode Island | 1961-1970 |  |  |  |  |  |  |  |
| Rhode Island | 1971-1980 |  |  |  |  |  |  |  |
| Rhode Island | 1800-1940 |  |  |  |  |  |  |  |
| Rhode Island | 1980-2000 |  |  |  |  |  |  |  |
| Rhode Island | 2000-2023 |  |  |  |  |  |  |  |
| South Carolina | 1941-1950 |  |  |  |  |  |  |  |
| South Carolina | 1951-1960 |  |  |  |  |  |  |  |
| South Carolina | 1961-1970 |  |  |  |  |  |  |  |
| South Carolina | 1971-1980 |  |  |  |  |  |  |  |
| South Carolina | 1800-1940 |  |  |  |  |  |  |  |
| South Carolina | 1980-2000 |  |  |  |  |  |  |  |
| South Carolina | 2000-2023 |  |  |  |  |  |  |  |
| South Dakota | 1941-1950 |  |  |  |  |  |  |  |
| South Dakota | 1951-1960 |  |  |  |  |  |  |  |
| South Dakota | 1961-1970 |  |  |  |  |  |  |  |
| South Dakota | 1971-1980 |  |  |  |  |  |  |  |
| South Dakota | 1800-1940 |  |  |  |  |  |  |  |
| South Dakota | 1980-2000 |  |  |  |  |  |  |  |
| South Dakota | 2000-2023 |  |  |  |  |  |  |  |
| Tennessee | 1941-1950 |  | Reported |  |  |  |  | [13] |
| Tennessee | 1951-1960 | Reported |  |  |  |  |  | [13] |
| Tennessee | 1961-1970 |  |  |  |  |  |  |  |
| Tennessee | 1971-1980 |  |  |  |  |  |  |  |
| Tennessee | 1800-1940 |  | Reported |  | Reported |  |  | [5, 32] |
| Tennessee | 1980-2000 |  |  |  |  |  |  |  |
| Tennessee | 2000-2023 |  |  |  |  | Reported |  | [47] |
| Texas | 1941-1950 |  |  |  |  |  |  |  |
| Texas | 1951-1960 |  |  |  |  |  |  |  |
| Texas | 1961-1970 |  |  |  |  |  |  |  |
| Texas | 1971-1980 |  |  |  |  |  |  |  |
| Texas | 1800-1940 |  |  |  |  |  |  |  |
| Texas | 1980-2000 |  |  |  |  |  |  |  |
| Texas | 2000-2023 |  |  |  |  |  |  |  |
| Utah | 1941-1950 | Reported |  |  |  |  |  | [9, 27] |
| Utah | 1951-1960 | Reported |  |  |  |  |  | [14] |
| Utah | 1961-1970 | Reported | Reported | Reported |  |  |  | [18, 48] |
| Utah | 1971-1980 | Reported | Reported | Reported |  |  |  | [48, 49] |
| Utah | 1800-1940 |  |  |  |  |  |  |  |
| Utah | 1980-2000 |  | Reported | Reported |  |  |  | [49] |
| Utah | 2000-2023 |  |  |  |  |  |  |  |
| Vermont | 1941-1950 |  |  |  |  |  |  |  |
| Vermont | 1951-1960 |  |  |  |  |  |  |  |
| Vermont | 1961-1970 |  |  |  |  |  |  |  |
| Vermont | 1971-1980 |  |  |  |  |  |  |  |
| Vermont | 1800-1940 |  |  |  |  |  |  |  |
| Vermont | 1980-2000 |  |  |  |  |  |  |  |
| Vermont | 2000-2023 |  |  |  |  |  |  |  |
| Virginia | 1941-1950 | Reported |  |  | Reported |  |  | [12, 26] |
| Virginia | 1951-1960 | Reported |  |  |  |  |  | [13, 14] |
| Virginia | 1961-1970 |  |  |  |  |  |  |  |
| Virginia | 1971-1980 |  |  |  |  |  |  |  |
| Virginia | 1800-1940 | Reported |  |  | Reported |  |  | [32, 50] |
| Virginia | 1980-2000 |  |  |  |  |  |  |  |
| Virginia | 2000-2023 |  |  |  |  |  |  |  |
| Washington | 1941-1950 |  |  |  |  |  |  |  |
| Washington | 1951-1960 |  |  |  |  |  |  |  |
| Washington | 1961-1970 |  |  |  |  |  |  |  |
| Washington | 1971-1980 |  |  |  |  |  |  |  |
| Washington | 1800-1940 |  | Reported |  |  |  |  | [5] |
| Washington | 1980-2000 |  |  |  |  |  |  |  |
| Washington | 2000-2023 |  |  |  |  |  |  |  |
| West Virginia | 1941-1950 |  |  |  |  |  |  |  |
| West Virginia | 1951-1960 |  |  |  |  |  |  |  |
| West Virginia | 1961-1970 |  |  |  |  |  |  |  |
| West Virginia | 1971-1980 |  |  |  |  |  |  |  |
| West Virginia | 1800-1940 |  |  |  |  |  |  |  |
| West Virginia | 1980-2000 |  |  |  |  |  |  |  |
| West Virginia | 2000-2023 |  |  |  |  |  |  |  |
| Wisconsin | 1941-1950 |  |  |  |  |  |  |  |
| Wisconsin | 1951-1960 |  |  |  |  |  |  |  |
| Wisconsin | 1961-1970 |  |  |  |  |  |  |  |
| Wisconsin | 1971-1980 |  |  |  |  |  |  |  |
| Wisconsin | 1800-1940 |  |  |  |  |  |  |  |
| Wisconsin | 1980-2000 |  |  |  |  |  |  |  |
| Wisconsin | 2000-2023 |  |  |  |  |  |  |  |
| Wyoming | 1941-1950 |  |  |  |  |  |  |  |
| Wyoming | 1951-1960 |  |  |  |  |  |  |  |
| Wyoming | 1961-1970 |  |  |  |  |  |  |  |
| Wyoming | 1971-1980 |  |  |  |  |  |  |  |
| Wyoming | 1800-1940 |  |  |  |  |  |  |  |
| Wyoming | 1980-2000 |  |  |  |  |  |  |  |
| Wyoming | 2000-2023 |  |  |  |  |  | Reported | [51] |

**REFERENCES FOR SUPPLEMENTARY DATA**

1. Rausch R. Recent Studies on Hydatid Disease in Alaska. Parassitologia. 1960;2 3:391-8.

2. Wilson JF, Diddams AC, Rausch RL. Cystic hydatid disease in Alaska: a review of 101 autochthonous cases of Echinococcus granulosus infection. American Review of Respiratory Disease. 1968;98 1:1-15.

3. Pinch L, Wilson J. Non-surgical management of cystic hydatid disease in Alaska: a review of 30 cases of Echinococcus granulosus infection treated without operation. Annals of Surgery. 1973;178 1:45.

4. Rausch R. Hydatid disease in boreal regions. Arctic. 1952;5 3:157-74.

5. Magath TB. The importance of sylvatic hydatid disease. Journal of the American Veterinary Medical Association. 1954;125 932:411-4.

6. Castrodale LJ, Beller M, Wilson JF, Schantz PM, McManus DP, Zhang L-H, et al. Two atypical cases of cystic echinococcosis (Echinococcus granulosus) in Alaska, 1999. The American journal of tropical medicine and hygiene. 2002;66 3:325-7.

7. Nakao M, McManus DP, Schantz PM, Craig PS, Ito A. A molecular phylogeny of the genus Echinococcus inferred from complete mitochondrial genomes. Parasitology. 2007;134 Pt 5:713-22; doi: 10.1017/S0031182006001934. <https://www.ncbi.nlm.nih.gov/pubmed/17156584>.

8. Jenkins EJ, Castrodale LJ, de Rosemond SJ, Dixon BR, Elmore SA, Gesy KM, et al. Tradition and transition: parasitic zoonoses of people and animals in Alaska, northern Canada, and Greenland. Advances in parasitology. 2013;82:33-204.

9. Pappaioanou M, Schwabe CW, Sard DM. An evolving pattern of human hydatid disease transmission in the United States. Am J Trop Med Hyg. 1977;26 4:732-42; doi: 10.4269/ajtmh.1977.26.732. <https://www.ncbi.nlm.nih.gov/pubmed/329700>.

10. Schantz PM, Alstine CV, Blacksheep A, Sinclair S. Prevalence of Echinococcus granulosus and other cestodes in dogs on the Navajo reservation in Arizona and New Mexico. Am J Vet Res. 1977;38 5:669-70. <https://www.ncbi.nlm.nih.gov/pubmed/879566>.

11. SCHANTZ PM. Echinococcosis in American Indians living in Arizona and New Mexico: a review of recent studies. American Journal of Epidemiology. 1977;106 5:370-9.

12. Franklin M, Ward J. Echinococcus infection in Mississippi. A new record of a natural infection in dogs. Journal of Parasitology. 1953;39 5.

13. Hutchison WF. Studies on the hydatid worm, Echinococcus granulosus, in Mississippi. Tulane University; 1958.

14. KATZ AM, PAN CT. Echinococcus disease in the United States. Am J Med. 1958;25 5:759-70; doi: 10.1016/0002-9343(58)90014-7. <https://www.ncbi.nlm.nih.gov/pubmed/13582986>.

15. Sawitz W. Echinococcus infection in Louisiana. The Journal of Parasitology. 1938;24 5:437-9.

16. Brunetti OA, Rosen MN. Prevalence of Echinococcus granulosus hydatid in California deer. The Journal of Parasitology. 1970:1138-40.

17. Tucker HA. Hydatid disease at the Los Angeles County Hospital, 1936-1948; with a report of first autochthonous case from California. Amer J Trop Med. 1951;31 1:83-9.

18. Sawyer JC, Schantz PM, Schwabe CW, Newbold MW. Identification of transmission foci of hydatid disease in California. Public Health Rep. 1969;84 6:531-41. <https://www.ncbi.nlm.nih.gov/pubmed/4977534>.

19. Liu IK, Schwabe CW, Schantz PM, Allison MN. The occurrence of Echinococcus granulosus in coyotes (Canis latrans) in the central valley of California. J Parasitol. 1970;56 6:1135-7. <https://www.ncbi.nlm.nih.gov/pubmed/5534027>.

20. Schantz PM, Clérou RP, Liu IK, Schwabe CW. Hydatid disease in the central valley of California. Transmission of infection among dogs, sheep, and man in Kern county. Am J Trop Med Hyg. 1970;19 5:823-30; doi: 10.4269/ajtmh.1970.19.823. <https://www.ncbi.nlm.nih.gov/pubmed/5465773>.

21. Miller CW, Ruppanner R, Schwabe CW. Hydatid disease in California. Study of hospital records, 1960 through 1969. Am J Trop Med Hyg. 1971;20 6:904-13. <https://www.ncbi.nlm.nih.gov/pubmed/5167186>.

22. Araujo F, Schwabe C, Sawyer J, Davis W. Hydatid disease transmission in California: a study of the Basque connection. American Journal of Epidemiology. 1975;102 4:291-302.

23. Ruppanner R, Schwabe CW. Early records of hydatid disease in California. American Journal of Tropical Medicine and Hygiene. 1973;22 4:485-92.

24. Cerda JR, Buttke DE, Ballweber LR. Echinococcus spp. Tapeworms in North America. Emerg Infect Dis. 2018;24 2:230-5; doi: 10.3201/eid2402.161126. <https://www.ncbi.nlm.nih.gov/pubmed/29350139>.

25. Passarelli P, Ramchandar N, Naheedy J, Kling K, Choi L, Pong A. AN 8-YEAR-OLD CALIFORNIA GIRL WITH ASYMPTOMATIC HEPATIC CYSTS. Pediatr Infect Dis J. 2022;41 7:e295-e6; doi: 10.1097/INF.0000000000003539. <https://www.ncbi.nlm.nih.gov/pubmed/35421052>.

26. Meltzer H, Kovacs L, Orford T, Matas M. Echinococcosis in North American indians and eskimos. Canadian Medical Association Journal. 1956;75 2:121.

27. Carlquist JH, Dowell RJ. Echinococcus Disease :  Report Of Four Cases Contracted In The United States. Rocky  Mountain  Medical  Journal. 1951;46:773 - 6.

28. Cerda JR, Ballweber LR. Confirmation of Echinococcus canadensis G8 and G10 in Idaho Gray Wolves ( Canis lupus) and Cervids. J Wildl Dis. 2018;54 2:403-5; doi: 10.7589/2017-05-119. <https://www.ncbi.nlm.nih.gov/pubmed/29369720>.

29. Foreyt WJ, Drew ML, Atkinson M, McCauley D. Echinococcus granulosus in gray wolves and ungulates in Idaho and Montana, USA. J Wildl Dis. 2009;45 4:1208-12; doi: 10.7589/0090-3558-45.4.1208. <https://www.ncbi.nlm.nih.gov/pubmed/19901399>.

30. LYON IP. A review of echinococcus disease in North America. The American Journal of the Medical Sciences (1827-1924). 1902;123 1:124.

31. Swartzwelder J. Echinococcus infection (hydatid disease) in Louisiana. Journal of Parasitology. 1946;32 Suppl.

32. Haight C, Alexander J. Hydatid cysts of the lung. Archives of Internal Medicine. 1940;65 3:510-23.

33. Lichtenwalner A, Adhikari N, Kantar L, Jenkins E, Schurer J. Echinococcus granulosus genotype G8 in Maine moose (Alces alces). Alces: A Journal Devoted to the Biology and Management of Moose. 2014;50:27-33.

34. Schurer JM, Bouchard E, Bryant A, Revell S, Chavis G, Lichtenwalner A, et al. Echinococcus in wild canids in Québec (Canada) and Maine (USA). PLoS Negl Trop Dis. 2018;12 8:e0006712; doi: 10.1371/journal.pntd.0006712. <https://www.ncbi.nlm.nih.gov/pubmed/30125277>.

35. Erickson AB. Helminths of Minnesota Canidae in relation to food habits, and a host list and key to the species reported from North America. The American Midland Naturalist. 1944;32 2:358-72.

36. Mech D. The Wolves of Isle Royale .   Fauna of the National Parks of the United States, Fauna Series 7. vol. 7. Washington, D. C.: U. S. Government Printing Office; 1966.

37. Bernstein LA, Shaffer C, Walz E, Moore S, Sparks A, Stone S, et al. Exploring Risk for Echinococcosis Spillover in Northern Minnesota Tribal Communities. Ecohealth. 2021;18 2:169-81; doi: 10.1007/s10393-021-01547-7. <https://www.ncbi.nlm.nih.gov/pubmed/34508275>.

38. HUTCHISON WF, BRYAN MW. Studies on the hydatid worm, Echinococcus granulosus. I. Species identification of the parasite found in Mississippi. Am J Trop Med Hyg. 1960;9:606-11; doi: 10.4269/ajtmh.1960.9.606. <https://www.ncbi.nlm.nih.gov/pubmed/13717068>.

39. Hutchison WF. Studies on the hydatid worm, Echinococats granulosus. II. Prevalence in Mississippi. American Journal of Tropical Medicine and Hygiene. 1960;9 6:612-5.

40. Brooks TJ, Webb WR, Heard KM. Hydatid disease: a summary of human cases in Mississippi. AMA Archives of Internal Medicine. 1959;104 4:561-7.

41. Ward JW. Additional Records of Echinococcus Granulosus From Dogs in the Lower Mississippi Region. J Parasitol. 1965;51:552-3. <https://www.ncbi.nlm.nih.gov/pubmed/14339364>.

42. Daly JJ, McDaniel RC, Husted GS, Harmon H. Unilocular hydatid cyst disease in the mid-South. JAMA. 1984;251 7:932-3.

43. Ramsey J. Echinococcus: Presence in wolves, and agency efforts on public education and outreach. 2010.

44. AlSalman A, Mathewson A, Martin IW, Mahatanan R, Talbot EA. Cystic Echinococcosis in Northern New Hampshire, USA. Emerg Infect Dis. 2023;29 5:1057-8; doi: 10.3201/eid2905.221828. <https://www.ncbi.nlm.nih.gov/pubmed/37044131>.

45. Katz R, Murphy S, Kosloske A. Pulmonary echinococcosis: A pediatric disease of the Southwestern United States. Pediatrics. 1980;65 5:1003-6.

46. Jensen LA, Short J, Andersen F: **Internal parasites of Odocoileus hemionus of central Utah**. In: *Proc Helminthol Soc Wash1982*: 317-9.

47. Dell B, Newman SJ, Purple K, Miller B, Ramsay E, Donnell R, et al. Retrospective investigation of Echinococcus canadensis emergence in translocated elk (Cervus canadensis) in Tennessee, USA, and examination of canid definitive hosts. Parasit Vectors. 2020;13 1:330; doi: 10.1186/s13071-020-04198-9. <https://www.ncbi.nlm.nih.gov/pubmed/32605660>.

48. KLOCK LE, SPRUANCE SL, ANDERSEN FL, JURANEK DD, KAGAN IG. Detection of asymptomatic hydatid disease by a community screening program. American journal of epidemiology. 1973;97 1:16-21.

49. Andersen FL, Crellin JR, Nichols CR, Schantz PM. Evaluation of a Program to Control Hydatid Disease in Central Utah. The Great Basin Naturalist. 1983;43 1:65-72.

50. Johnston Jr JH, Twente GE. Pulmonary Hydatid (Echinococcic) Cyst: Report of Native Case. Annals of Surgery. 1952;136 2:305.

51. Pipas MJ, Fowler DR, Bardsley KD, Bangoura B. Survey of coyotes, red foxes and wolves from Wyoming, USA, for Echinococcus granulosus s. l. Parasitol Res. 2021;120 4:1335-40; doi: 10.1007/s00436-021-07059-1. <https://www.ncbi.nlm.nih.gov/pubmed/33521842>.
